# Computational Prediction of Immunogenic Epitopes and Protein Structures of Heat Shock Protein 90 in *Toxoplasma gondii*

**DOI:** 10.1002/iid3.70584

**Published:** 2026-11-06

**Authors:** Amirhossein Abedi, Ali Dalir Ghaffari, Sobhan Dehghanpour, Mohammad Fekrikia

**Affiliations:** ^1^ Student Research Committee, Faculty of Medicine Shahed University Tehran Iran; ^2^ Department of Parasitology and Mycology, Faculty of Medicine Shahed University Tehran Iran

**Keywords:** epitope prediction, Hsp90, immunoinformatics, *Toxoplasma gondii*, vaccine design

## Abstract

**Introduction:**

*Toxoplasma gondii* (*T. gondii*) is an obligate intracellular protozoan parasite that poses a significant threat to both human and animal health worldwide. Despite extensive research efforts, no effective vaccine is currently available for human use. Heat shock protein 90 (Hsp90) is a highly conserved molecular chaperone essential for protein folding, signal transduction, and stress response regulation in *T. gondii*. Due to its immunological importance and developmental regulation, Hsp90 is a promising target for vaccine development.

**Material and Methods:**

In the present study, comprehensive immunoinformatics approaches were employed to characterize *T. gondii* Hsp90. Various bioinformatics tools were used to analyze its physicochemical properties, subcellular localization, post‑translational modifications, structural features, and potential immunogenic epitopes.

**Results:**

The protein comprised 708 amino acids, had a molecular weight of 81.93 kDa, and was predicted to be unstable, soluble, and nonallergenic. Structural modeling and validation revealed that 50.85% of the residues form α‐helices, and 15.68% β‐strands, with two disulfide bonds contributing to conformational stability. Multiple bioinformatics tools identified several B‐ and T‐cell epitopes with high antigenicity, strong binding affinity to both MHC Class I and II molecules, and favorable immunogenic profiles. The peptide SEDLPLNISRESLQQNKILK obtained the highest predicted B‐cell epitope score using SVMTriP, while ANMERIMKAQALRDN was selected as a promising T‐cell epitope based on MHC‐II binding prediction and VaxiJen antigenicity assessment. The overall antigenicity scores (0.757 for ANTIGENpro and 0.551 for VaxiJen) confirmed Hsp90 as an immunogenic and safe vaccine candidate.

**Conclusion:**

The findings highlight *T. gondii* Hsp90 as a promising target for subunit vaccine development, providing a theoretical foundation for future in vitro and in vivo validation studies.

## Graphical abstract

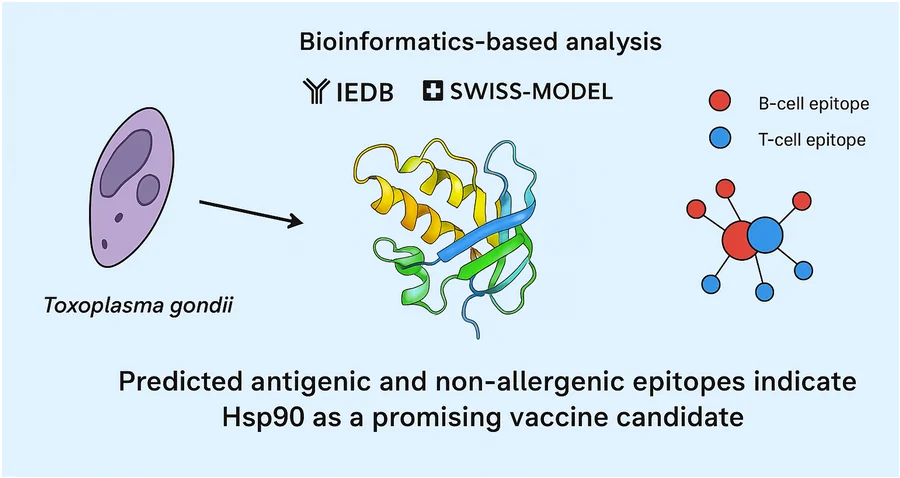

## 1 Introduction

*Toxoplasma gondii* (*T. gondii*) is an opportunistic parasite belonging to the phylum Apicomplexa [1]. Its sexual cycle occurs in felids, which serve as the definitive hosts, and its asexual cycle occurs in human body and a wide range of warm‑blooded intermediate hosts [2]. Humans primarily acquire infection via ingesting undercooked meat containing tissue cysts, or through ingestion of food or water contaminated with sporulated oocysts [3]. A key feature of the parasite's life cycle is the interconversion between rapidly replicating tachyzoites and slowly dividing bradyzoites that persist within tissue cysts. Tachyzoites drive acute disease and, in congenital infection, can cause neurological and ocular disease, whereas bradyzoites can persist lifelong and reactivate in immunocompromised hosts [4, 5, 6]. Current chemotherapeutic regimens mainly target tachyzoites and do not eradicate tissue cysts; adverse effects (e.g., hypersensitivity, bone‐marrow suppression) may further limit their use [7]. Accordingly, given the latent infection worldwide burden and reactivation risk, vaccine design remains a priority for both human and veterinary health. Rational antigen selection should prioritize conserved, surface‐exposed, or secreted proteins with low human homology, appropriate MHC presentation and population coverage, and favorable safety profiles [8]. In recent years, various vaccination strategies against *T. gondii* have been investigated, including live‑attenuated vaccines, recombinant protein vaccines, and DNA vaccines [9]. Among these approaches, DNA vaccines may protect against toxoplasmosis by stimulating long‐lasting humoral and cell‐associated immunity in animal models, thus helping to provide protection against parasitic infections [10, 11]. As a result, creating a vaccine for the prevention of toxoplasmosis is an attractive avenue for preventing both infection and disease [12]. Although durable protection has been demonstrated in livestock, there is currently no licensed human vaccine for *T. gondii* [13]. The Heat Shock Protein (HSP) families consist of a diverse array of proteins that are consistently produced in large amounts and whose expression rises in response to stressful conditions [14]. Notably, Hsp90 plays a crucial role in both stressed and unstressed cells, serving as a chaperone that manages essential processes related to cell growth and differentiation. It coordinates protein homeostasis, refolding misfolded proteins, promoting maturation, facilitating intracellular trafficking, regulating nuclear hormone receptors and other transcription factors, orchestrating kinase signaling, and controlling translation [15, 16]. Given that Hsp90 expression and subcellular localization in *T. gondii* are developmentally regulated, particularly during tachyzoite–bradyzoite conversion, this chaperone represents a promising immunological target for vaccine design [17].

Bioinformatics is a well‐established and rapidly advancing interdisciplinary field that integrates mathematical, statistical, and computational methods to analyze biological data. This includes the prediction of epitopes, necessary for determining the immunogenicity of antigens in vaccine development. Bioinformatics tools and online platforms allow researchers to detect possible B‐ and T‐cell epitopes, thus improving the accuracy of vaccine candidate development [18, 19, 20, 21]. Also, this method is employed to assess gene and protein expression, predict protein structures and physicochemical properties, and evaluate immunogenicity. In silico methods, based on computer simulations rather than laboratory experiments, enable the prediction of antigenic proteins even without in vitro expression [22]. So, this study characterizes the *T. gondii* Hsp90 protein using immunoinformatics: predicting structural features, evaluating antigenicity, and identifying cytotoxic T‐lymphocyte (CTL), putative B‐cell, and helper T‐lymphocyte (HTL) epitopes to inform vaccine design.

## 2 Materials and Methods

### 2.1 Amino Acid Sequence Retrieval

The *T. gondii* Hsp90 amino acid sequence (Accession Number: A0A125YVB7) was resourced in FASTA format via UniProtKB.

### 2.2 Prediction of Physico‐Chemical Functions

Expasy ProtParam predicted the Hsp90's physico‐chemical properties, demonstrating molecular weight (MW), the number of amino acids, instability index, extinction coefficients, the grand average of hydropathicity (GRAVY), protein isoelectric point (pI) and half‐life in vivo and in vitro, whole count of residues having negative and positive charges, instability index, and aliphatic index [23].

### 2.3 Post‐Translational Modification (PTM)

NetPhos 3.1 identified tyrosine, threonine, and serine phosphorylation sites. SignalP determined the sequence's signal peptide. The protein possesses a Lipoprotein signal peptide (Sec/SPII), no signal peptide (Other), a Tat signal peptide (Tat/SPI) or a Sec signal peptide (Sec/SPI) [24]. Additionally, the NetAcet‐1.0 was utilized to identify potential acylation areas in the Hsp90 sequence [25]. Additionally, NetNGlyc 1.0 and NetOGlyc 4.0 forecasted N‐ and O‐linked glycosylation areas [26, 27].

### 2.4 Predicting Subcellular Localization and Transmembrane Domains

DeepLoc‐2.1 forecasted in silico subcellular localization and DEEP‐TMHMM predicted the presence of transmembrane helices.

### 2.5 Predicting Tertiary and Secondary Structures

The secondary structure of Hsp90 was predicted using PSIPRED, which employs position‐specific scoring matrices generated by PSI‐BLAST to predict α‐helices, β‐strands, and coils [28]. Additional secondary structure analyses were performed using the GOR‐IV [29] and the NetSurfP3 [30] Servers. Predicted disulfide bonds (cysteine pairs) were identified using the DIpro online server. Furthermore, the three‐dimensional structure of Hsp90 was modeled using the SWISS‐MODEL server [31].

### 2.6 Tertiary Structure Refinement and Confirmation

Of the SWISS‐MODEL‐ created templates and models, one that exhibited proper coverage and sequence identity performed refinement using Galaxy Refine, employing the CASP10‐tested approach [32, 33]. It is capable of rearranging side chains, repacking them, and relaxing the whole structure via molecular dynamics simulations. SWISS‐MODEL produced Ramachandran plots to assess the tertiary structure validity [34]. The “Ramachandran plot is utilized to predict the likelihood of a particular amino acid forming secondary structures based on the dihedral angles *ɸ* and *Ψ* (allowed and disallowed) of amino acids, which are calculated based on the van der Waals radius of side chain atoms” [35].

### 2.7 Forecasting Linear and Conformational B‐Cell Epitopes

ABCpred identified the linear B‐cell epitopes [36]. Additionally, BcePred forecasted consecutive B‐cell epitopes (accuracy: 58.7%), taking account physico‐chemical features, including turns, exposed area, antigenic propensity, accessibility, polarity, hydrophilicity, and flexibility [37, 38]. Furthermore, BCPREDS 1.0 predicted B‐cell epitopes (default specificity: 75%; 20‐mer epitopes) [39], and SVMTriP estimated the B‐cell epitopes considering the Propensity and Tripeptide correlation values [40]. ProtScale generated the linear epitopes graphical representation, utilizing beta‐turn, hydrophobicity, average flexibility, the accessible residues' percentage, and alpha‐helix [23]. ElliPro identified discontinuous B‐cell epitopes based on the PDB file, using a maximum distance of 6 angstroms and a default minimum score of 0.5 [41]. Finally, the VaxiJen 2.0, AlgPred [42], and PepCalc respectively assessed epitopes antigenicity, allergenicity, and water solubility.

### 2.8 Forecasting Major Histocompatibility Complex (MHC)‐Specific Epitopes

The method recommended by the Immune Epitope Database (IEDB) predicted the IC_50_ scores for peptides attached to MHC Class II and I molecules. MHC‐I‐specific epitopes associated with the mouse alleles H2‐Ld, H2‐Dd, H2‐Db, H2‐Kb, H2‐Kd, and H2‐Kk were predicted. Also, MHC‐II epitopes were predicted for mouse alleles H2‐IAd, H2‐IEd, and H2‐IAb. The predictions for peptides containing 10 amino acids were carried out using the IEDB‐recommended approach. Following this, the immunogenicity of the identified epitopes was assessed using IEDB MHC Class I [43]. It followed the IEDB guidelines, and the anticipated peptide length was fixed at 15 amino acids and organized by percentile rank. Subsequently, VaxiJen 2.0, IFNepitope, and IL4‐pred predicted the potential and antigenicity for inducing IL‐4 and IFN‐γ for each epitope, employing hybrid methods. Murine MHC alleles were selected as an initial model for epitope screening, which is commonly used in preclinical immunoinformatics studies before validation using human HLA alleles.

### 2.9 Forecasting CTL Epitopes

CTLpred analyzed and forecasted CTL epitopes by a combined technique having 75.8% accuracy. The cutoff points for artificial neural network and support vector machine were fixed at 0.36 and 0.51, respectively [44]. Then, IEDB MHC Class I forecasted the epitopes' immunogenicity [43].

### 2.10 Solubility, Antigenicity, and Allergenicity Evaluation

Finally, ANTIGENpro [45] and VaxiJen 2.0 [46] assessed Hsp90's antigenicity. ANTIGENpro forecasts antigenicity solely using microarray data, without utilizing pathogen or alignment values. However, VaxiJen applies an innovative alignment‐free method that converts sequences into vectors of principal amino acid properties through auto‐cross covariance. VaxiJen's predictions have an accuracy of 70%–89%, considering the targeted organism (https://www.ddg-pharmfac.net/vaxijen/VaxiJen/VaxiJen.html). Also, allergenicity predictions were made by AlgPred, utilizing the MEME/MAST motif [42]. SOLpro predicted the protein solubility following overexpression [47].

## 3 Results

### 3.1 General Properties of the *T. gondii* Hsp90 Gene

This protein has 708 amino acid residues (hypothesized pI: 4.96; MW: 81.93319 kDa). It is composed of 3633 carbon, 5776 hydrogen, 950 nitrogen, 1142 oxygen, and 28 sulfur. It has 108 positively (Arg + Lys) and 143 negatively charged residues (Asp + Glu). There are a total of 11,529 atoms within the sequence, and shows an extinction coefficient of 68,675 M^−1^ cm^−1^ in water at 280 nm. Its half‐life is > 10 h (*Escherichia coli* [*E. coli*], in vivo), > 20 h (yeast, in vivo), and 30 h (mammalian reticulocytes, in vitro). The instability index score is 43.98. Furthermore, it exhibited an aliphatic index of 81.50, with a GRAVY value of −0.626. The complete ProtParam‐derived physicochemical output for TgHsp90 (UniProt accession: A0A125YVB7) is provided as Table S1 to improve transparency and reproducibility.

### 3.2 Predicting PTM Sites of Hsp90

According to NetAcet‐1.0 and NetPhos 3.1, there is 1 acetylation site and 67 phosphorylation areas in the Hsp90 protein sequence, including 14 tyrosine, 23 threonine, and 30 serine residues (Figure 1). SignalP predicted Hsp90's putative signal peptide. It had a Sec signal peptide (Sec/SPI) with a likelihood of 0.0003, while the probability of other peptides is 0.9997 (Figure 2). Additionally, Hsp90 has four N‐linked (Figure 3) and one O‐linked glycosylation sites.

**Figure 1:**
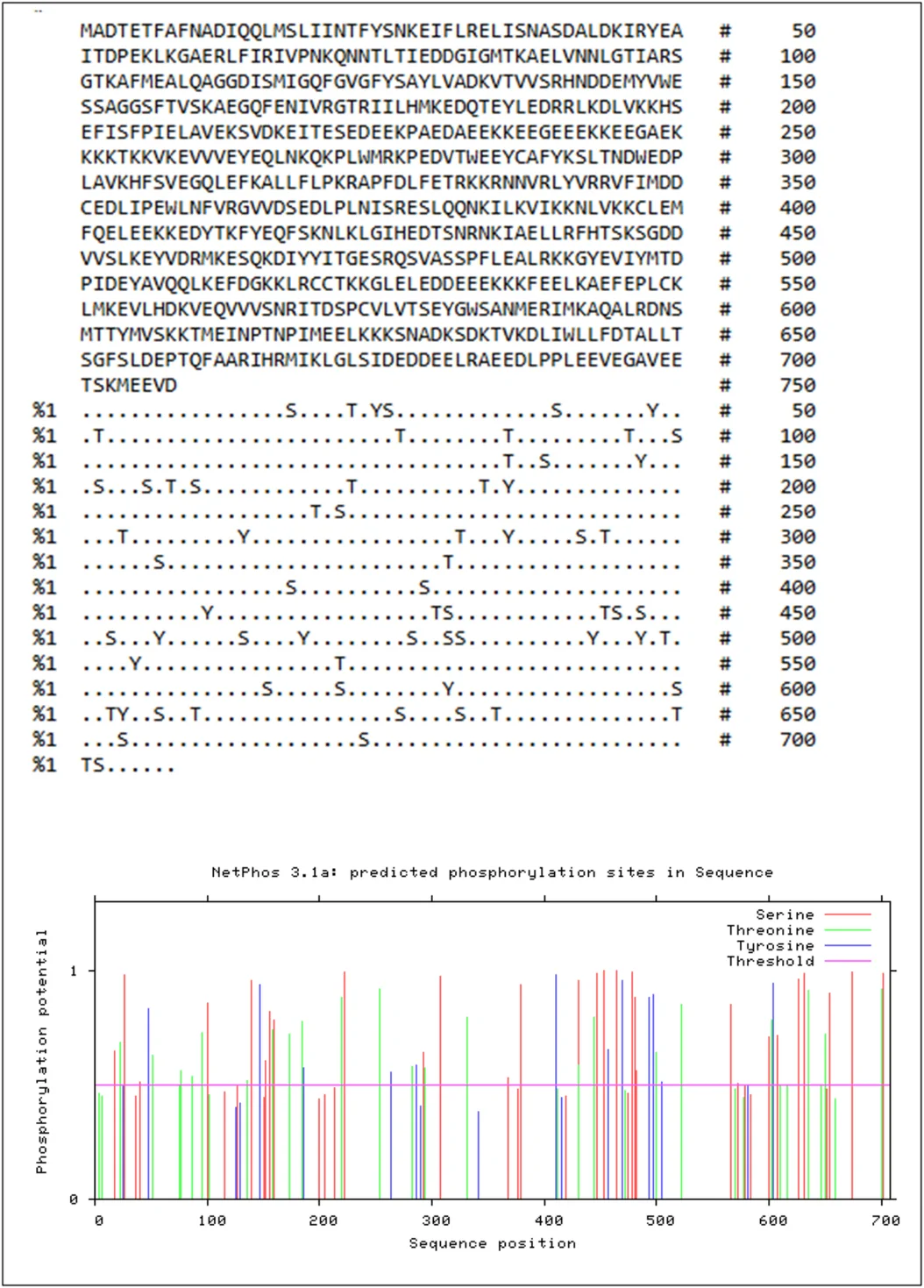
The output from the NetPhos server for phosphorylation sites of Hsp90.

**Figure 2:**
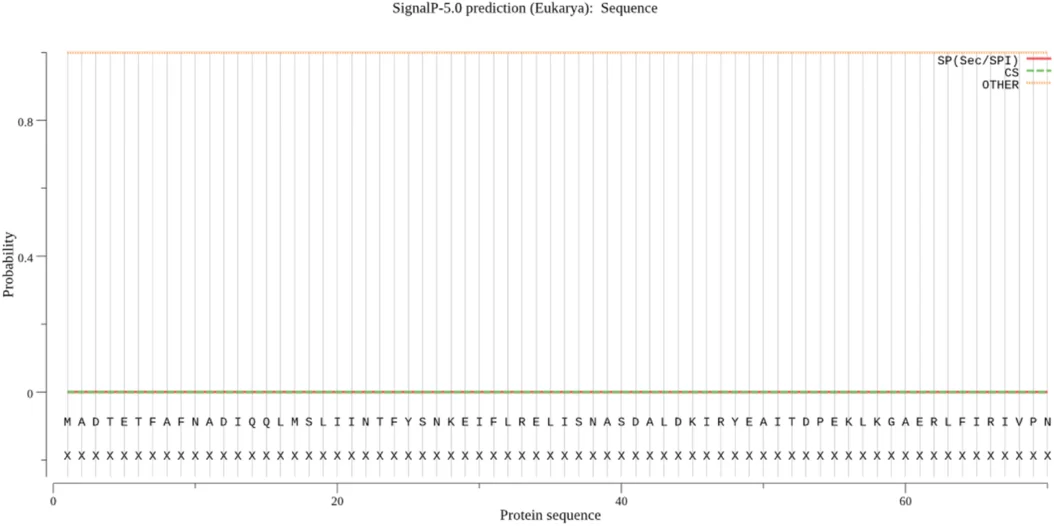
Signal peptide prediction of the Hsp90 protein of *T. gondii*, using the SignalP‐5.0 online tool. Other: the probability that the sequence does not have any kind of signal peptide.

**Figure 3:**
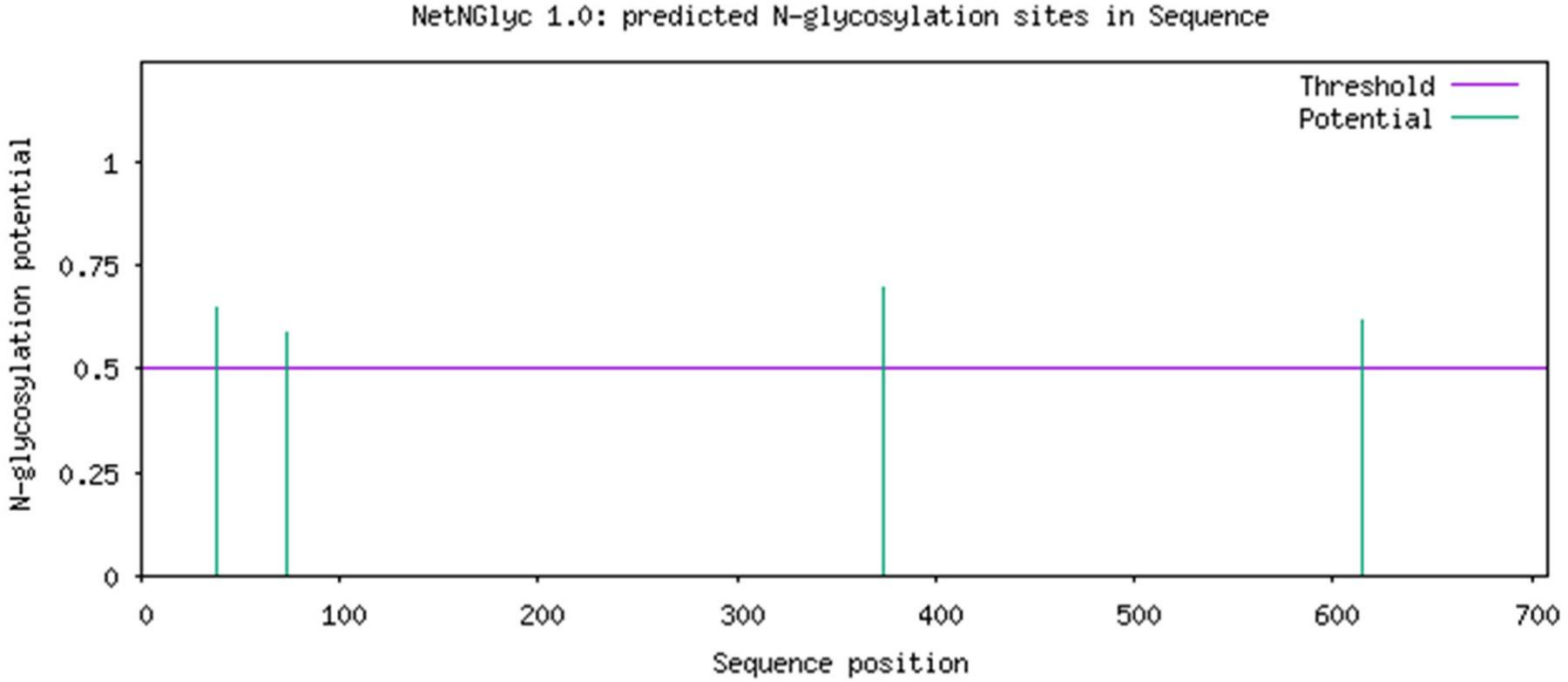
The output from the NetNGlyc server for N‐glycosylation sites of Hsp90.

### 3.3 Predicting Transmembrane Domains and Subcellular Localization of Hsp90 Protein

The subcellular localization of the target protein was predicted using DeepLoc‐2.1. The protein is most likely located in the cytoplasm, with a probability score of 0.7623. It is also predicted to be in the nucleus (0.3040) and the Golgi apparatus (0.3342) as less likely sites. Additionally, the protein is predicted to be soluble (probability: 0.8890), with low chances of being peripheral (0.4170), lipid‐anchored (0.2610), or transmembrane (0.0240) associated with membranes. Interestingly, the analysis also detected a nuclear export signal (NES), suggesting it may shuttle between the nucleus and cytoplasm. Furthermore, the DEEP‐TMHMM server results indicated the absence of a transmembrane domain within the Hsp90 sequence (Figure 4).

**Figure 4:**
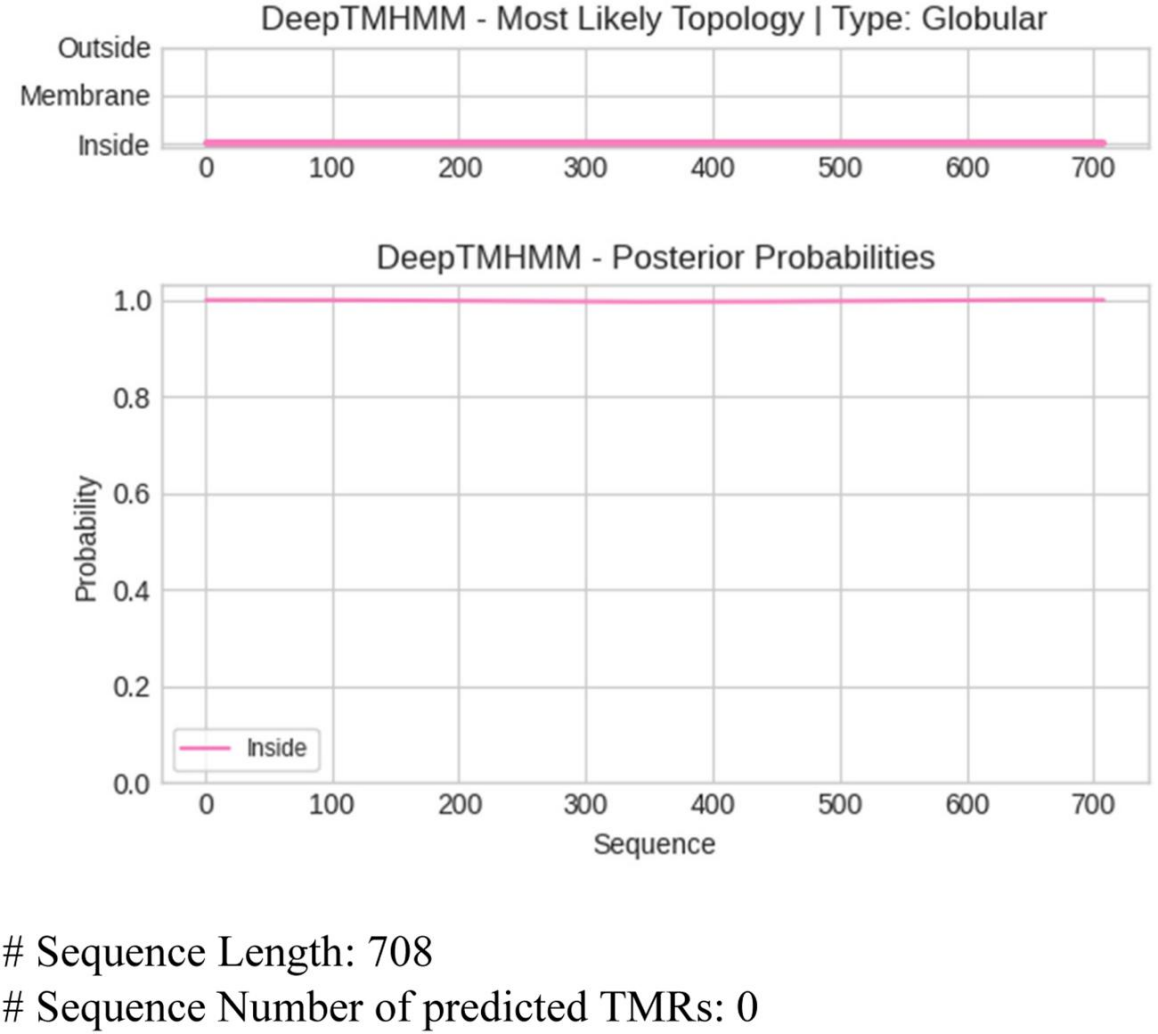
Prediction of transmembrane topology and localization of the Hsp90 protein using the DEEPTMHMM.

### 3.4 Tertiary and Secondary Structure Analysis

GOR IV, PSIPRED, and NetSurfP3 forecasted Hsp90's secondary structure elements, like extended strand, alpha helix, and random coil (Figures 5, 6, 7). GOR IV secondary structure prediction identified 237 (33.47%) random coil, 360 (50.85%) alpha helix, and 111 (15.68%) extended strand. Additionally, the analysis found two cysteine residues in Hsp90, forming disulfide bonds at certain sites, like residues 287–351 and 549–574 (Table 1). The Hsp90 model was generated using SWISS‐MODEL. We selected the model with 82.98% sequence identity and high coverage. Figure 8 indicates the SWISS‐MODEL results.

**Figure 5:**
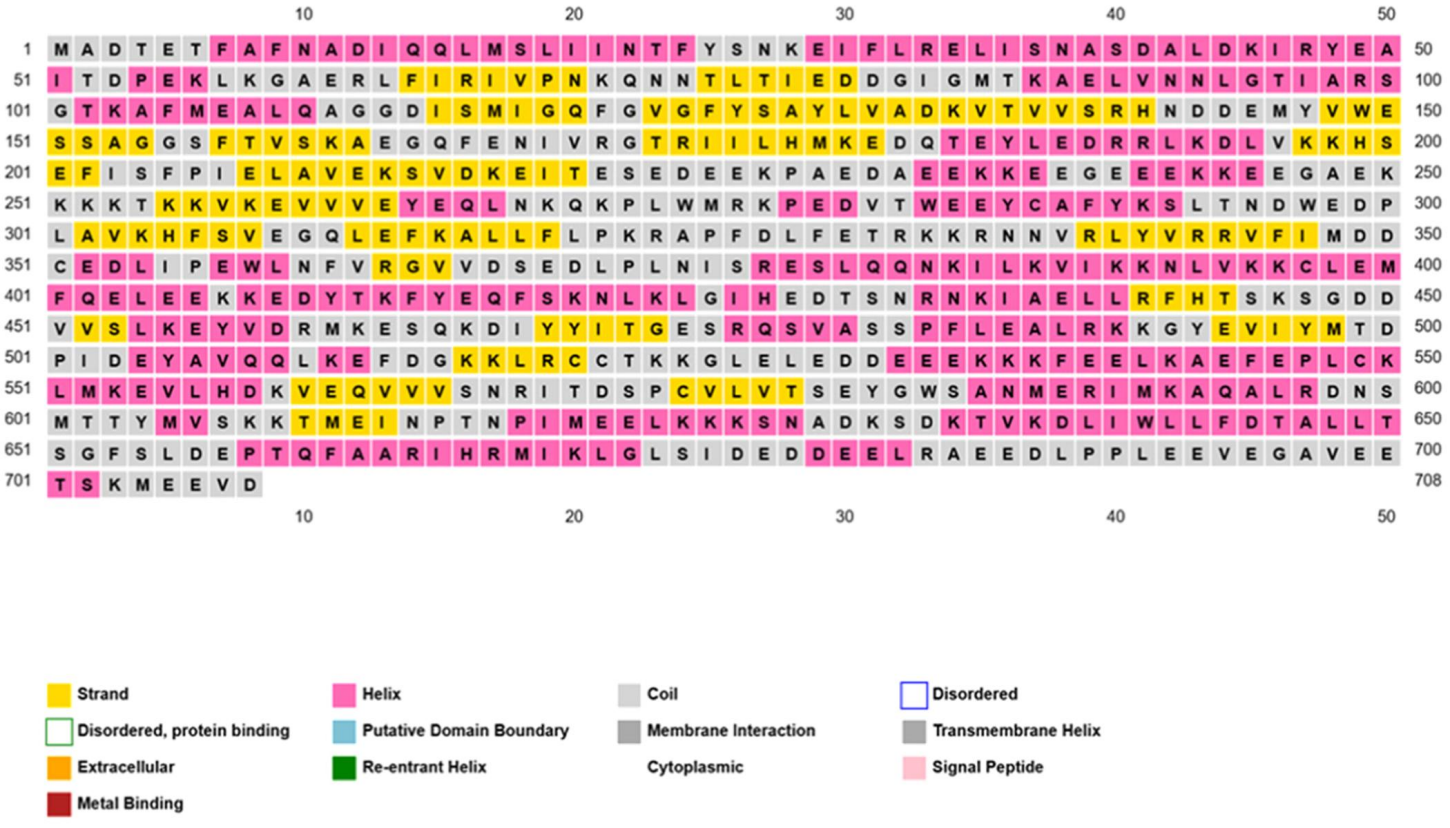
Graphical result from secondary structure prediction of the vaccine using PSIPRED.

**Figure 6:**
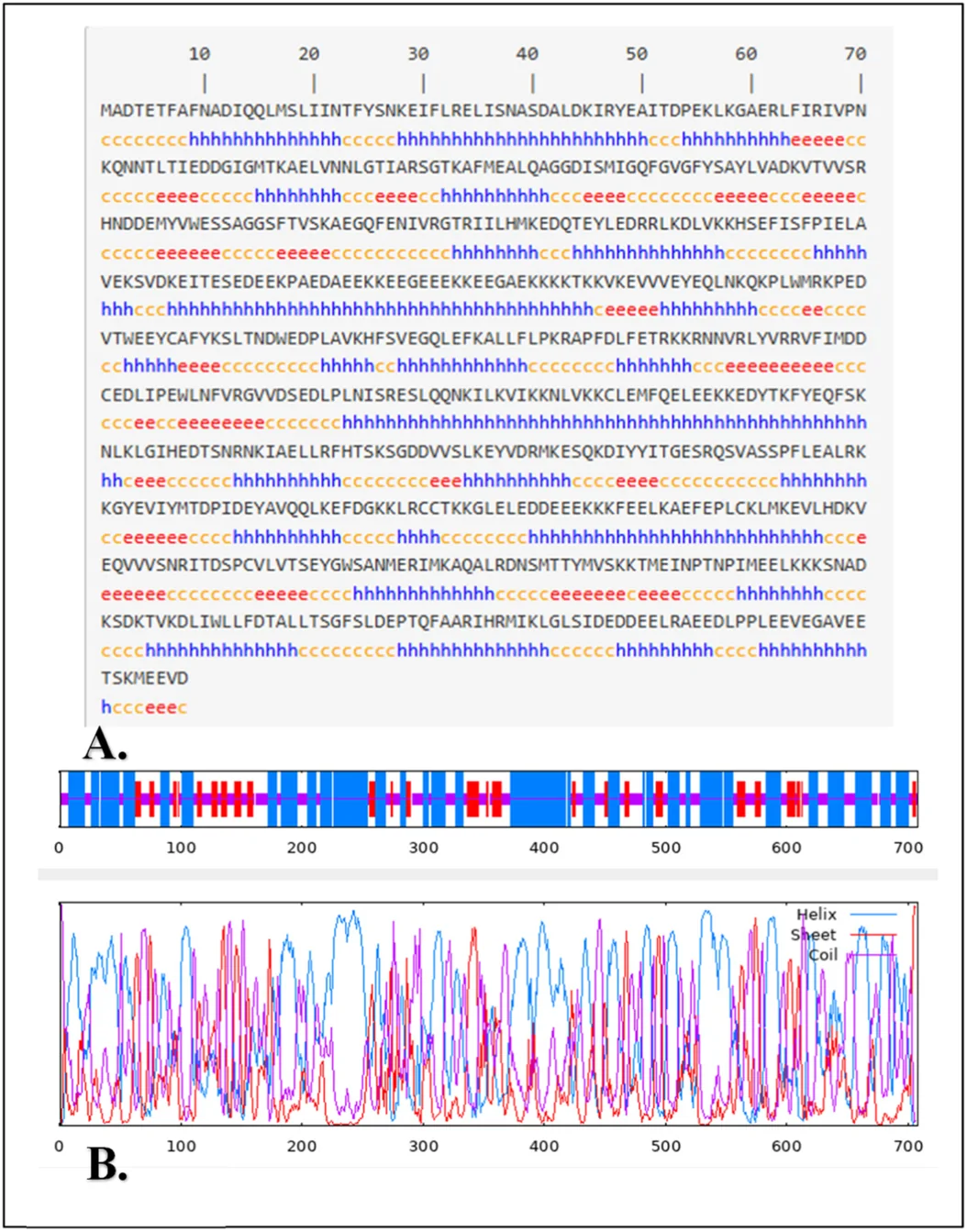
(A) Predicted secondary structure of Hsp90 using GOR IV. (B) Graphical representation of the predicted secondary structure of *T. gondii* Hsp90 protein. h, helix; e, extended strand; c, coil.

**Figure 7:**
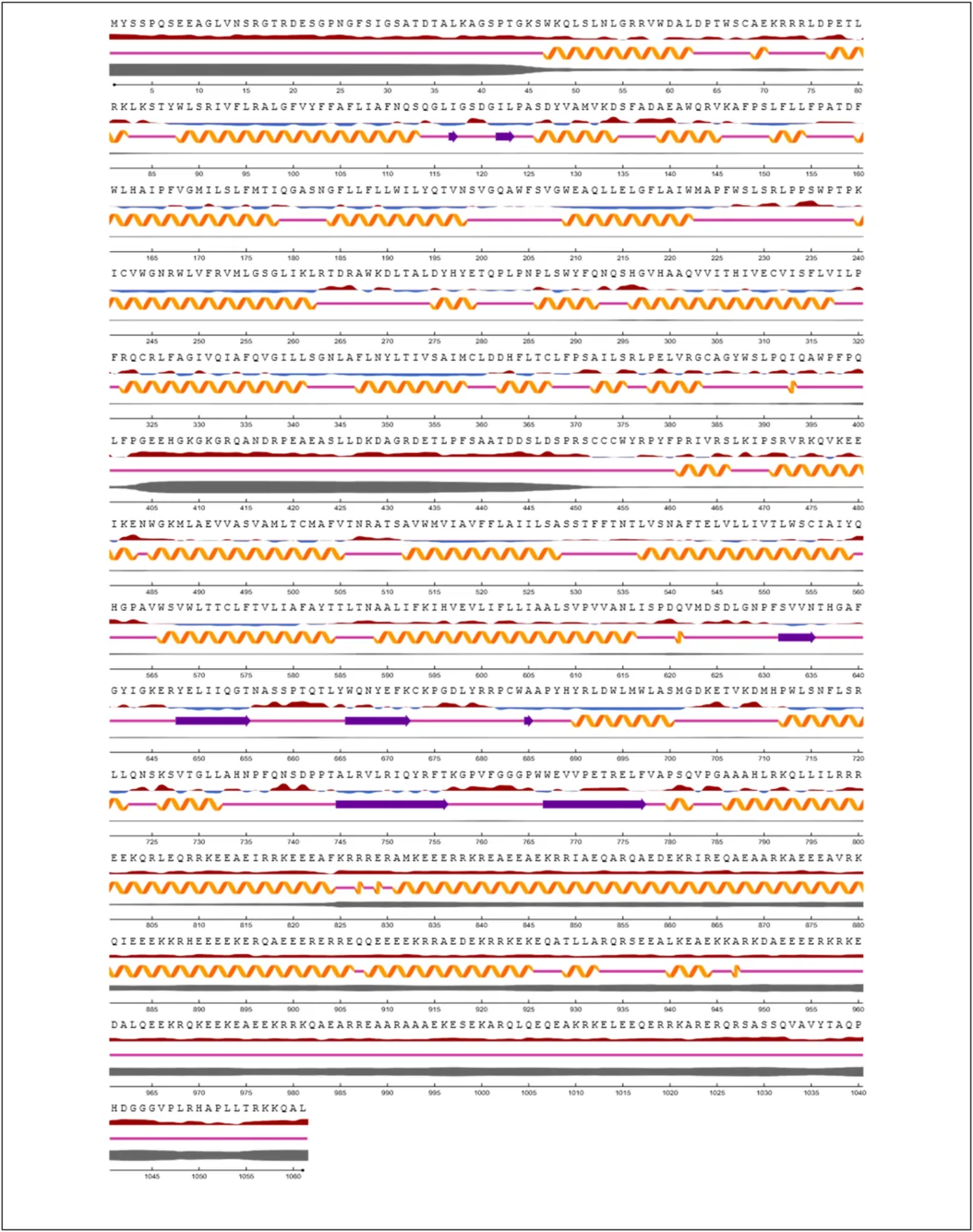
Predicted secondary structure of the protein obtained using NetSurfP3.0.

**Table 1:** Disulfide bonds predicted using DIpro.

Bond index	Cys1_Position	Cys2_Position
1	287	351
2	549	574

**Figure 8:**
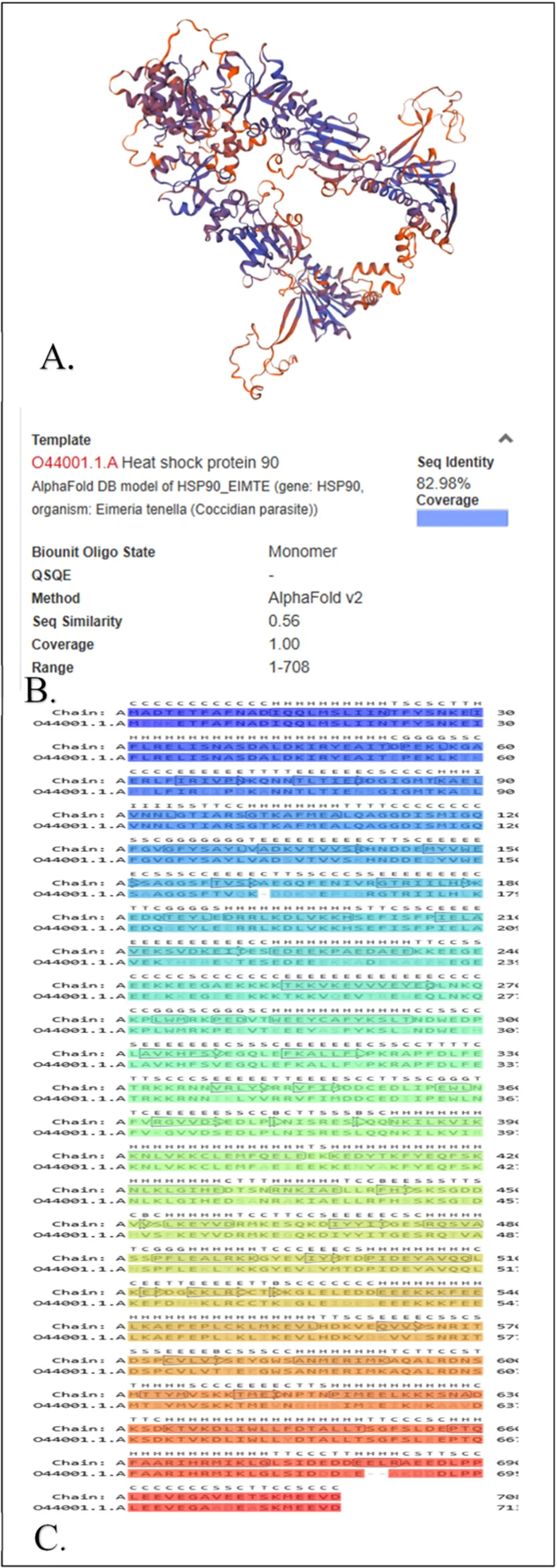
Output of the SWISS‐MODEL server. (A) Computed 3D model. (B) Identity, similarity, and coverage of sequence. (C) Model‐template alignment.

### 3.5 Refinement and Validation of the Tertiary Structure

GalaxyRefine performed the refining process, indicating Model 2 as the most successfully refined template based on GDT‐HA (0.9700), Clash score (8.6), RMSD (0.387), Poor rotamers (0.3%), MolProbity (1.464), and Rama favored (99.0%). ProSA‐web evaluated the model quality, reporting a *Z*‐score of −9.67 for the refined model and −9.41 for the crude model (Figure 9A,B). Also, 96.18% of residues were in the favored site, and 0.99% were in Ramachandran outlier sites. After refining, the favored site included 98.6% of residues (Figure 9C,D).

**Figure 9:**
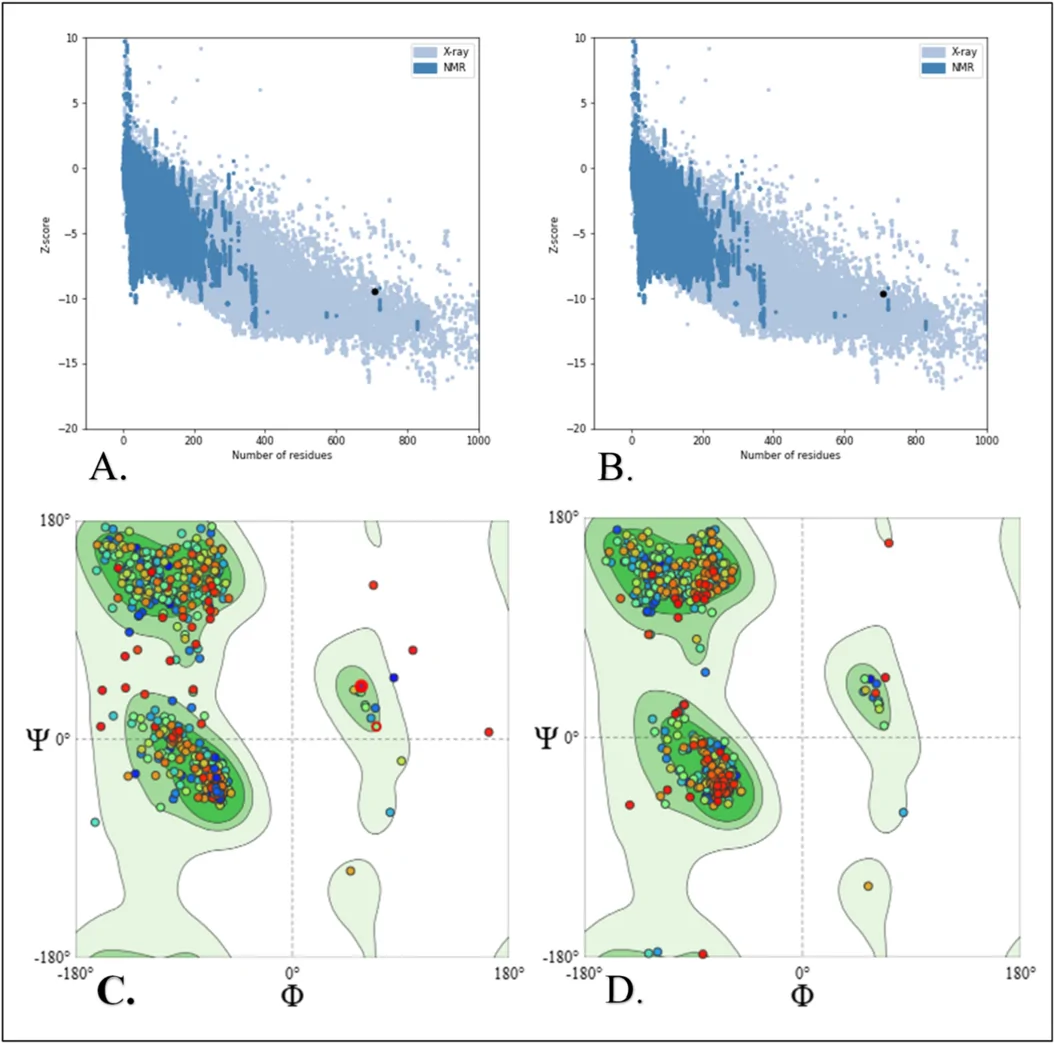
Validation of the 3D model of Hsp90 protein using Ramachandran plot. The *z*‐score plot for the 3D structure of the predicted vaccine before and after refinement with the ProSA‐web server (A and B). The analysis of the Ramachandran plot for the initial model and after refinement is shown in (C and D).

### 3.6 Linear and Conformational B Cell Epitopes

ABCpred predicted linear B‐cell epitopes that are 16 amino acids long, exhibiting higher scores indicating higher forecasting reliability (Table 2). Allergenicity analysis using AlgPred indicated that the majority of the predicted ABCpred‐derived B‐cell epitopes were classified as potential allergens. However, a limited number of epitopes, including RVFIMDDCEDLIPEWL and GFSLDEPTQFAARIHR, were predicted to be nonallergenic while retaining favorable antigenicity and solubility, making them more attractive candidates for subsequent vaccine development. Also, ProtScale and BcePred identified B‐cell‐specific epitopes (Figure 10 and Table 3). BCPREDs and SVMTriP results are shown in Tables 4 and 5. The mean scores for Bepipred linear epitope anticipation, beta‐turn, flexibility, antigenicity, hydrophilicity, and surface accessibility of Hsp90 calculated with IEDB were 0.938, 1.010, 1.855, 0.091, 1.000, and 1.006, respectively (Figure 11). While forecasting conformational B cell epitopes with ElliPro, 12 discontinuous B cell epitopes were found: 4 residues (score: 0.994), 7 residues (0.985), 10 residues (0.93), 9 residues (0.896), 6 residues (0.865), 36 residues (0.859), 122 residues (0.714), 10 residues (0.712), 128 residues (0.683), 6 residues (0.591), 6 residues (0.530), and 5 residues (0.503) (Table 6 and Figure 12).

**Table 2:** Predicted B‐cell epitope using the ABCpred server.

Rank	Sequence	Start position	Score	VaxiJen score	Allergenicity	Water solubility
1	DVTWEEYCAFYKSLTN	280	0.96	−0.2861	Allergen	Good
2	YEVIYMTDPIDEYAVQ	493	0.95	0.3896	Allergen	Good
3	GGDISMIGQFGVGFYS	112	0.93	0.6507	Allergen	Good
4	HFSVEGQLEFKALLFL	305	0.90	1.5007	Allergen	Good
5	TLTIEDDGIGMTKAEL	75	0.88	0.3222	Allergen	Poor
5	ASDALDKIRYEAITDP	39	0.88	0.4602	Allergen	Good
5	EEGEEEKKEEGAEKKK	237	0.88	1.1369	Allergen	Good
6	AERLFIRIVPNKQNNT	60	0.87	0.9105	Allergen	Good
7	DKEITESEDEEKPAED	216	0.86	1.3290	Allergen	Poor
8	DGIGMTKAELVNNLGT	81	0.85	−0.6482	Allergen	Good
8	TMEINPTNPIMEELKK	610	0.85	0.4676	Allergen	Good
9	VVSNRITDSPCVLVTS	564	0.84	0.0811	Allergen	Good
9	KGLELEDDEEEKKKFE	524	0.84	0.7993	Allergen	Good
9	QLNKQKPLWMRKPEDV	266	0.84	0.1426	Allergen	Good
9	TVSKAEGQFENIVRGT	158	0.84	0.5993	Allergen	Good
9	VWESSAGGSFTVSKAE	148	0.84	0.0602	Allergen	Good
10	TNPIMEELKKKSNADK	616	0.83	0.7374	Allergen	Good
10	TYMVSKKTMEINPTNP	603	0.83	0.2533	Allergen	Poor
10	EDLIPEWLNFVRGVVD	352	0.83	0.1989	Allergen	Poor
10	RVFIMDDCEDLIPEWL	344	0.83	0.6818	aNonallergen	Good
11	TNDWEDPLAVKHFSVE	294	0.82	0.4647	Allergen	Good
11	MKEDQTEYLEDRRLKD	179	0.82	1.0443	Allergen	Good
11	AYLVADKVTVVSRHND	128	0.82	0.4977	Allergen	Good
12	GFSLDEPTQFAARIHR	652	0.81	1.1371	aNonallergen	Good
12	SPCVLVTSEYGWSANM	572	0.81	0.5600	Allergen	poor
12	KIRYEAITDPEKLKGA	45	0.81	0.8340	Allergen	Good
12	KRAPFDLFETRKKRNN	322	0.81	−0.6101	Allergen	Good
13	VVSLKEYVDRMKESQK	451	0.80	0.8317	Allergen	Good
13	EDAEEKKEEGEEEKKE	230	0.80	1.5049	Allergen	Good

^a^ Nonallergenic epitopes with favorable antigenicity and solubility are considered priority candidates for future experimental validation.

**Figure 10:**
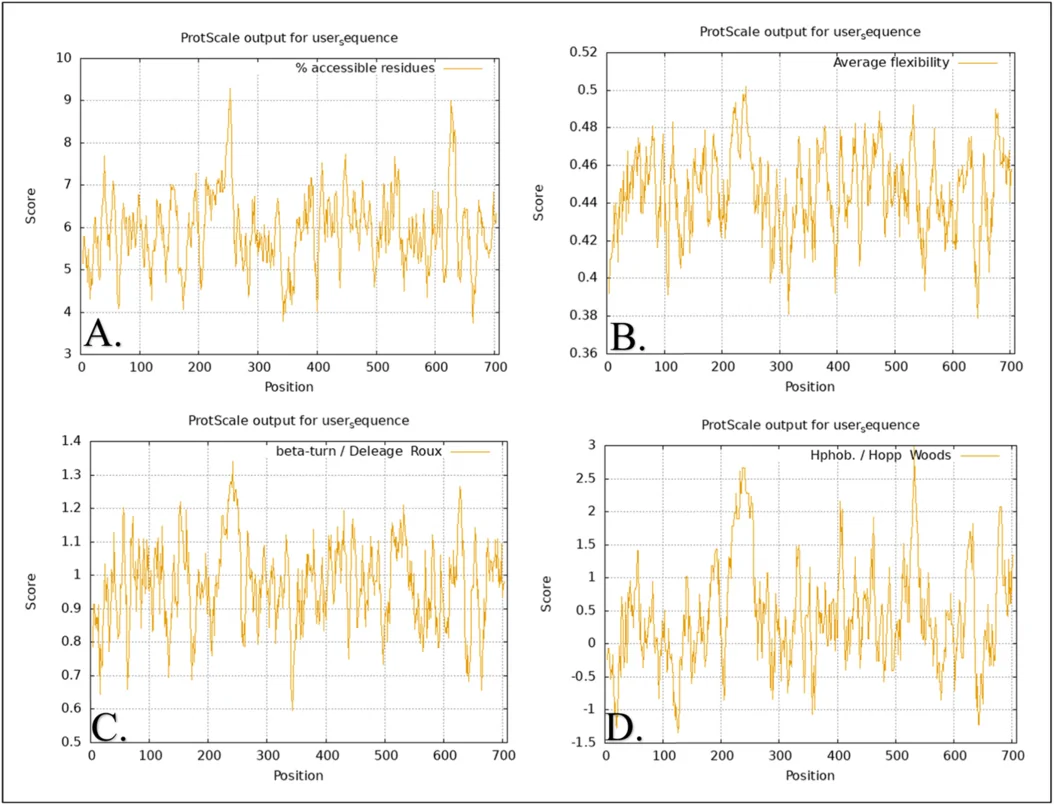
Linear B‐cell epitopes of Hsp90 protein sequence based on (A) percent of accessible residues, (B) average flexibility, (C) beta turn, and (D) hydrophobicity.

**Table 3:** The predicted B‐cell epitopes, using physico‐chemical properties based on the BcePred server.

Hydrophilicity	PNKQNNT, ARSGTKA, VSRHNDDEM, ESSAGGS, HMKEDQTEY, EDRRLKD, EKSVDKEITESEDEEKPAEDAEEKKEEGEEEKKEEGAEKKKKTKKVKE, RKPEDVT, TNDWEDPETRKKRNN, GVVDSED, QELEEKKEDYTK, HEDTSNRN, KHTSKSGDDVVS, DRMKESQKDI, TGESRQSV, KEFDGKK, ELEDDEEEKKKFEE, SNRITDS, RDNSMTT, KKKSNADKSDKTVKD, SIDEDDEELRA, EGAVEETSKMEE
Flexibility	RIVPNKQN, GTIARSGT, TVVSRHN, YVWESSAG, FTVSKAE, LHMKEDQ, EYLEDRRL, LAVEKSVDKEITESEDEE, PAEDAEEKKEEGEEEKKEEGAEKKKKTKKV, FDLFETRKKRNN, LPLNISRES, QELEEKK, GIHEDTSNRN, LRFHTSKSGD, EYVDRMKESQ, YYITGESRQ, KEFDGKK, LELEDDEEEKKKF, YMVSKKT, PIMEELKKKSNADKSDKT, LSIDEDD
Accessibility	NTFYSNKEIF, LDKIRYEAITDPEKLKGAER, RIVPNKQNNTLT, VSRHNDDEMYV, KAEGQFE, LHMKEDQTEYLEDRRLKDLVKKHSEFI, VEKSVDKEITESEDEEKPAEDAEEKKEEGEEEKKEEGAEKKKKTKKVKEVVVEYEQLNKQKPLWMRKPEDVTWEEY, YKSLTNDWEDPL, EGQLEFK, PKRAPFDLFETRKKRNNVRLY, SRESLQQNKILKVIKKNLVKK, FQELEEKKEDYTKFYEQFSKNLK, IHEDTSNRNKIAEL, RFHTSKSGDD, LKEYVDRMKESQKDIYYITGESRQSVA, LEALRKKGYEV, TDPIDEYAVQQLKEFDGKKLRC, KKGLELEDDEEEKKKFEELKAEFE, KEVLHDKVEQV, SNRITDSP, NMERIMKAQALRDNSMTTYMVSKKTMEINPTNPIMEELKKKSNADKSDKTVKDL, SLDEPTQFA, SIDEDDEELRAEEDLPPLEEVE, AVEETSKMEEVD
Turns	NKQNNTLT, VSRHNDDEM
Exposed surface	TDPEKLKG, VPNKQNNTL, SRHNDDE, MKEDQTEYLEDRRLKDLVKKH, EKSVDKE, ESEDEEKPAEDAEEKKEEGEEEKKEEGAEKKKKTKKVKEVV, EYEQLNKQKPLWMRKPEDVT, LFETRKKRNNVRL, ESLQQNKILKVIKKNLVKKC, QELEEKKEDYTKF, DTSNRNK, DRMKESQKDI, LEALRKKGYEV, KEFDGKK, ELEDDEEEKKKFEELK, KEVLHDK, SKKTMEIN, MEELKKKSNADKSDKTVKD, EDDEELR,
Polarity	, NKEIFLREL, DALDKIRYEAITDPEKLKGAERLFIRI, VSRHNDDEMY, KAEGQFE, RIILHMKEDQTEYLEDRRLKDLVKKHSEFIS, VEKSVDKEITESEDEEKPAEDAEEKKEEGEEEKKEEGAEKKKKTKKVKEVV, EYEQLNKQKPLWMRKPEDVTWEE, VKHFSVEGQLEFK, DLFETRKKRNNVRLYVRRV, IMDDCEDLI, LKVIKKNLVKKCLEMFQELEEKKEDYTKF, KLGIHEDTSNRNKIAELLRFHTSKS, LKEYVDRMKESQKDI, FLEALRKKGYEV, LKEFDGKKLRCCTKKGLELEDDEEEKKKFEELKAEFEPLCKLMKEVLHDKVEQV, NMERIMKAQ, VSKKTMEIN, PIMEELKKKSNADKSDKTVKDL, FAARIHRMIKLGLSIDEDDEELRAEEDLPPLEEVEGAVEETSKMEEVD
Antigenic propensity	, QLMSLII, IFLRELI, RLFIRIVP, DKVTVVSRH, KKVKEVVVEYEQL, VKHFSVE, LLFLPKR, NVRLYVRRVFI, LNFVRGVVDSEDL, KILKVIKKNLVKKCLE, GDDVVSLKEYV, GKKLRCCTKKGL, FEPLCKLMKEVLHDKVEQVVVSNRITDSPCVLVTSEY, VKDLIWLLFD, IKLGLSI, LPPLEEV,

**Table 4:** BCPred predictions of linear B‐cell epitopes from Hsp90 protein.

No.	Position	Epitope	Score	VaxiJen score	Allergenicity	Water solubility
1	230	EDAEEKKEEGEEEKKEEGAE	1	1.4851	Allergen	Good
2	681	ELRAEEDLPPLEEVEGAVEE	1	0.2862	Allergen	Good
3	523	KKGLELEDDEEEKKKFEELK	0.999	0.7409	Allergen	Good
4	251	KKKTKKVKEVVVEYEQLNKQ	0.997	0.6388	Allergen	Good
5	65	IRIVPNKQNNTLTIEDDGIG	0.993	0.7689	Allergen	Good
6	608	KKTMEINPTNPIMEELKKKS	0.986	0.4828	Allergen	Good
7	109	LQAGGDISMIGQFGVGFYSA	0.972	0.5634	Allergen	Poor
8	399	EMFQELEEKKEDYTKFYEQF	0.97	0.4797	Allergen	Good
9	175	IILHMKEDQTEYLEDRRLKD	0.969	1.0892	Allergen	Good
10	209	LAVEKSVDKEITESEDEEKP	0.969	0.6804	Allergen	Good
11	275	MRKPEDVTWEEYCAFYKSLT	0.96	0.1267	Allergen	Good
12	441	RFHTSKSGDDVVSLKEYVDR	0.928	0.4775	Nonallergen	Good
13	557	HDKVEQVVVSNRITDSPCVL	0.921	0.1856	Allergen	Good
14	329	FETRKKRNNVRLYVRRVFIM	0.915	1.3413	Allergen	Good
15	464	SQKDIYYITGESRQSVASSP	0.914	0.3027	Nonallergen	Good
16	134	KVTVVSRHNDDEMYVWESSA	0.91	0.5875	Allergen	Good
17	41	DALDKIRYEAITDPEKLKGA	0.819	0.2544	Allergen	Good
18	498	MTDPIDEYAVQQLKEFDGKK	0.776	0.0428	Allergen	Good

**Table 5:** B‐cell linear epitopes predicted using the SVMTriP server.

Rank	Location	Epitope	Score
1	368–387	SEDLPLNISRESLQQNKILK	1.000
2	583–602	WSANMERIMKAQALRDNSMT	0.858
3	292–311	SLTNDWEDPLAVKHFSVEGQ	0.737
4	548–567	LCKLMKEVLHDKVEQVVVSN	0.619
5	665–684	IHRMIKLGLSIDEDDEELRA	0.538
6	483–502	PFLEALRKKGYEVIYMTDPI	0.484
7	128–147	AYLVADKVTVVSRHNDDEMY	0.455
8	321–340	PKRAPFDLFETRKKRNNVRL	0.442
9	188–207	EDRRLKDLVKKHSEFISFPI	0.395
10	102–121	TKAFMEALQAGGDISMIGQF	0.311

**Figure 11:**
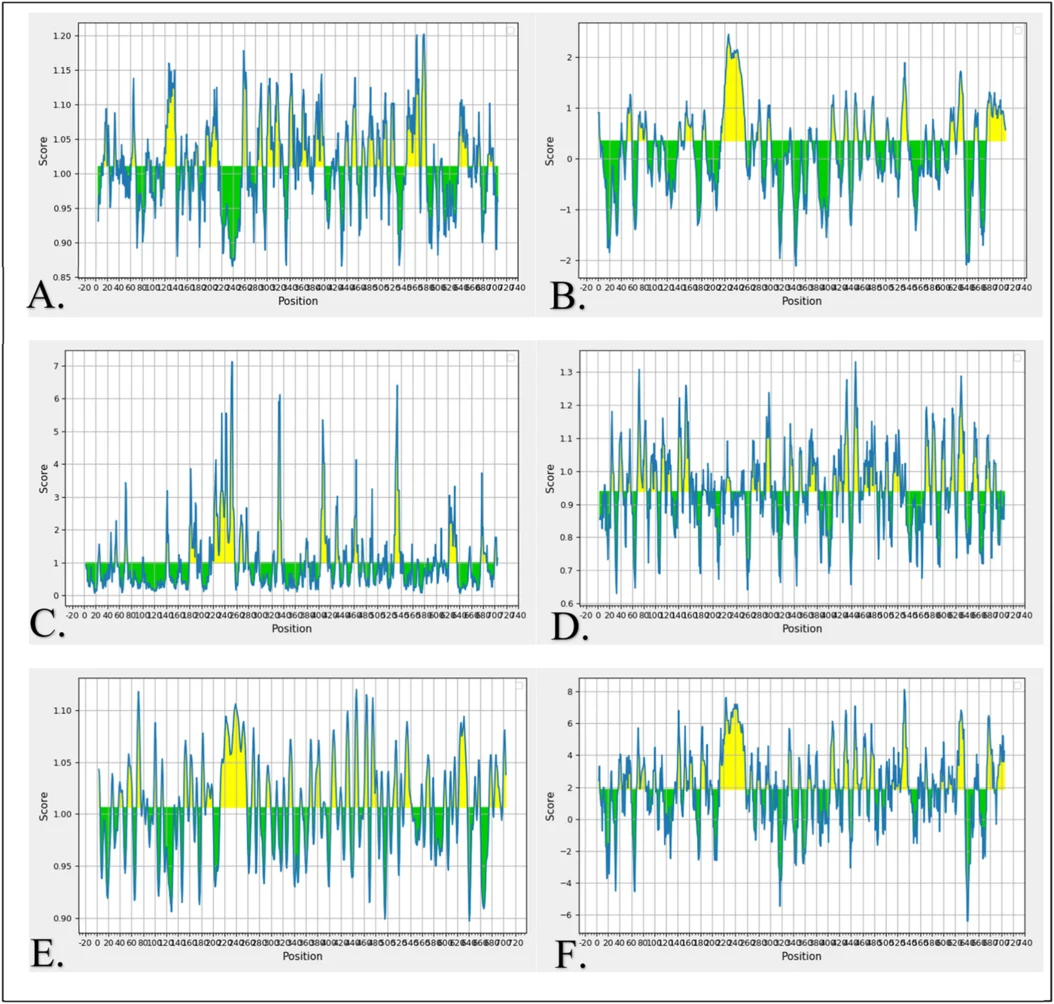
Propensity scale plots of Hsp90 protein. (A) Bepipred linear epitope prediction; (B) antigenicity; (C) beta‐turn; (D) surface accessibility; (E) hydrophilicity; (F) flexibility; on the graphs, the *Y*‐axis represents the corresponding score for each residue (averaged within the specified window), while the *X*‐axis represents the residue positions in the sequence. The tables provide values of calculated scores for each residue. The larger score for the residues might be interpreted as indicating that the residue has a higher probability of being part of the epitope (those residues are colored in yellow on the graphs). The green color (below the threshold) indicates unfavorable regions related to the properties of interest.

**Table 6:** Predicted discontinuous B‐cell epitope residues of the Hsp90 sequence.

No.	Residues	Number of residues	Score
1	A:G239, A:E240, A:E241, A:E242	4	0.994
2	A:K243, A:E245, A:E246, A:G247, A:A248, A:E249, A:K250	7	0.985
3	A:E677, A:D678, A:D679, A:E680, A:E681, A:L682, A:R683, A:A684, A:E685, A:E686	10	0.93
4	A:E692, A:E693, A:V694, A:E695, A:G696, A:A697, A:V698, A:E699, A:E700	9	0.896
5	A:T701, A:S702, A:K703, A:M704, A:E705, A:E706	6	0.865
6	A:E212, A:K213, A:S214, A:V215, A:D216, A:K217, A:E218, A:I219, A:T220, A:E221, A:S222, A:E223, A:D224, A:E225, A:E226, A:K227, A:P228, A:A229, A:E230, A:D231, A:A232, A:E233, A:E234, A:K235, A:K236, A:K253, A:T254, A:K255, A:K256, A:V257, A:K258, A:E259, A:V260, A:V261, A:V262, A:E263	36	0.859
7	A:V458, A:D459, A:M461, A:K462, A:E463, A:S464, A:Q465, A:D467, A:E486, A:A487, A:R489, A:K490, A:K491, A:G492, A:Y493, A:D514, A:G515, A:K516, A:K517, A:K536, A:K537, A:F538, A:E539, A:E540, A:L541, A:K542, A:A543, A:E544, A:F545, A:E546, A:P547, A:L548, A:C549, A:K550, A:L551, A:M552, A:K553, A:E554, A:V555, A:L556, A:H557, A:D558, A:K559, A:V560, A:E561, A:Q562, A:V563, A:V564, A:V565, A:S566, A:N567, A:R568, A:I569, A:T570, A:D571, A:S572, A:P573, A:C574, A:K608, A:M611, A:I613, A:N614, A:P615, A:T616, A:N617, A:P618, A:I619, A:M620, A:E621, A:E622, A:L623, A:K624, A:K625, A:K626, A:S627, A:N628, A:A629, A:D630, A:K631, A:S632, A:D633, A:K634, A:T635, A:V636, A:K637, A:D638, A:L639, A:I640, A:W641, A:L642, A:L643, A:F644, A:D645, A:T646, A:S654, A:L655, A:D656, A:E657, A:P658, A:T659, A:Q660, A:F661, A:A662, A:A663, A:R664, A:I665, A:H666, A:R667, A:M668, A:I669, A:K670, A:L671, A:G672, A:L673, A:S674, A:I675, A:D676, A:D687, A:L688, A:P689, A:P690, A:L691	122	0.714
8	A:L526, A:E527, A:L528, A:E529, A:D530, A:D531, A:E532, A:E533, A:E534, A:K535	10	0.712
9	A:M1, A:A2, A:D3, A:T4, A:E5, A:T6, A:F7, A:A8, A:F9, A:N10, A:A11, A:D12, A:I13, A:Q14, A:Q15, A:L16, A:M17, A:S18, A:L19, A:I20, A:I21, A:N22, A:T23, A:F24, A:K45, A:I46, A:Y48, A:E49, A:A50, A:I51, A:T52, A:D53, A:P54, A:E55, A:K56, A:L57, A:K58, A:G59, A:A60, A:E61, A:D81, A:G82, A:I83, A:G84, A:M85, A:T86, A:K87, A:A88, A:E89, A:L90, A:V91, A:N92, A:N93, A:L94, A:G95, A:T96, A:I97, A:A98, A:R99, A:S100, A:G101, A:T102, A:K103, A:A104, A:F105, A:M106, A:E107, A:A108, A:L109, A:Q110, A:A111, A:G112, A:G113, A:D114, A:I115, A:S116, A:M117, A:G119, A:Q120, A:G122, A:V123, A:Y126, A:Y129, A:L130, A:V131, A:A132, A:D133, A:K134, A:V135, A:T136, A:V137, A:V138, A:S139, A:R140, A:H141, A:N142, A:D143, A:D144, A:E145, A:M146, A:Y147, A:V148, A:W149, A:E150, A:S151, A:S152, A:A153, A:G154, A:G155, A:S156, A:F157, A:T158, A:V159, A:S160, A:K161, A:A162, A:E163, A:G164, A:Q165, A:F166, A:E167, A:N168, A:I169, A:V170, A:R171, A:H178, A:K180, A:D182	128	0.683
10	A:N70, A:K71, A:Q72, A:N73, A:N74, A:E181	6	0.591
11	A:R276, A:K277, A:P278, A:E279, A:D280, A:V281	6	0.53
12	A:E309, A:G310, A:Q311, A:L312, A:E313	5	0.503

**Figure 12:**
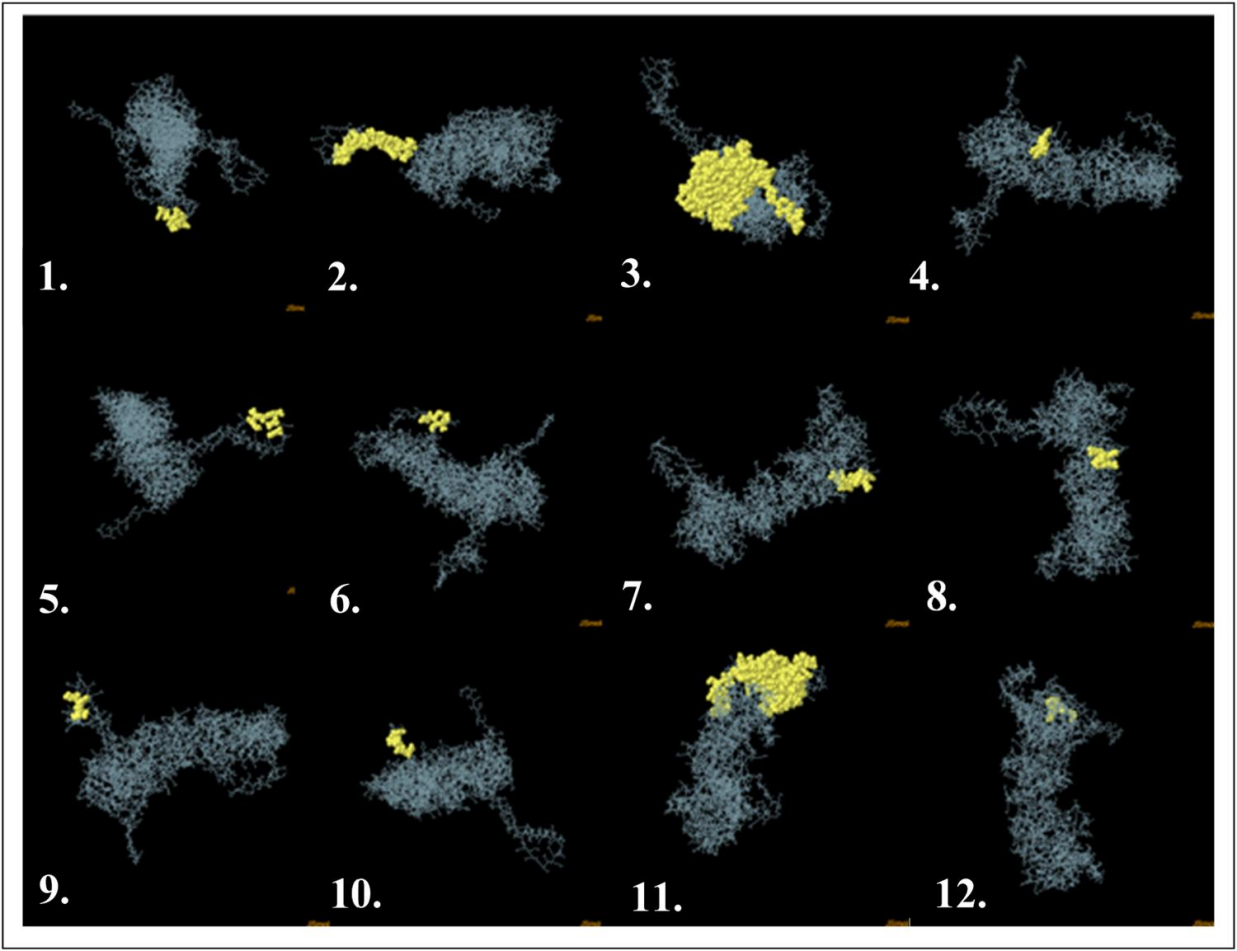
Predicted conformational B‐cell epitopes of *T. gondii* Hsp90 by ElliPro tool of IEDB Analysis Resource with best mode view.

### 3.7 Forecasting MHC‐Specific Epitopes

Tables 7 and 8 indicate the predicted MHC‐I (10‐mer) and MHC‐II (15‐mer) epitopes, based on the calculated IC50 values for peptide attached to alleles special for mice.

**Table 7:** Percentile rank for Hsp90 binding to MHC‐I molecules.

Allele	Start–end	Peptide sequence	Percentile rank	Immunogenicity
H‐2‐Db	48–57	YEAITDPEKL	0.14	0.15408
	430–439	TSNRNKIAEL	0.19	0.05048
	581–590	YGWSANMERI	0.24	−0.11333
H‐2‐Kb	413–422	KFYEQFSKNL	0.29	−0.24076
	26–35	SNKEIFLREL	0.64	0.36609
	578–587	TSEYGWSANM	0.92	0.15099
H‐2‐Kk	415–424	YEQFSKNLKL	0.23	−0.43235
	200–209	SEFISFPIEL	0.24	0.2852
	685–694	EEDLPPLEEV	0.31	0.107
H‐2‐Dd	67–76	IVPNKQNNTL	0.4	−0.32178
	58–67	KGAERLFIRI	1.1	0.40581
	26–35	SNKEIFLREL	1.7	0.36609
H‐2‐Kd	128–137	AYLVADKVTV	0.18	−0.43235
	602–611	TTYMVSKKTM	0.47	−0.66112
	23–32	TFYSNKEIFL	0.54	−0.109
H‐2‐Ld	371–380	LPLNISRESL	0.11	−0.00505
	320–329	LPKRAPFDLF	0.14	0.13182
	314–623	NPTNPIMEEL	0.23	0.10259

*Note:* Low percentile rank = good binders; IC_50_ values = percentile rank.

**Table 8:** Percentile rank for Hsp90 binding to MHC‐II molecules, antigenicity, IFN‐γ, and IL‐4 induction.

Allele	HTL epitope	Percentile rank	Antigenicity	IFN‐γ inducing		IL‐4 inducing	
				Result	Score	Result	SVM score
H2‐IAD	DKVEQVVVSNRITDS	1.10	0.3742	Positive	0.046	Positive	0.28
H2‐IAD	HDKVEQVVVSNRITD	1.40	0.4881	Negative	−0.256	Negative	0.18
H2‐IAD	ANMERIMKAQALRDN	1.40	0.7229	Negative	−0.263	Positive	0.27
H2‐IAB	LTNDWEDPLAVKHFS	0.59	0.4772	Negative	−0.338	Positive	0.29
H2‐IAB	TNDWEDPLAVKHFSV	0.59	0.3739	Negative	−0.286	Positive	0.28
H2‐IAB	SLTNDWEDPLAVKHF	0.84	0.3612	Positive	4.000	Positive	0.29
H2‐IED	RAPFDLFETRKKRNN	0.03	−0.4780	Positive	0.023	Positive	0.52
H2‐IED	ERLFIRIVPNKQNNT	0.08	1.0539	Negative	−0.269	Positive	0.58
H2‐IED	KRAPFDLFETRKKRN	0.10	−0.7268	Positive	0.067	Positive	0.58

### 3.8 CTL Epitope Forecasting

Considering the CTLpred findings, Table 9 shows the top 10 predicted 9‐mer CTL epitopes of Hsp90.

**Table 9:** Predicted Hsp90 epitopes by CTLpred.

Peptide rank	Start position	Sequence	Score (ANN/SVM)	Immunogenicity
1	169	IVRGTRIIL	0.94/1.2001383	0.3275
2	340	LYVRRVFIM	0.90/1.052005	0.3313
3	663	ARIHRMIKL	0.21/1.7324018	−0.05283
4	184	TEYLEDRRL	0.97/0.7104357	0.17994
5	660	QFAARIHRM	1.00/0.67091234	0.28529
6	60	AERLFIRIV	0.56/1.1051164	0.36636
7	593	AQALRDNSM	0.93/0.70631518	−0.0293
8	487	ALRKKGYEV	0.67/0.95183142	−0.32292
9	396	KCLEMFQEL	0.97/0.64428413	−0.00291
10	24	FYSNKEIFL	0.47/1.1149916	0.00476

### 3.9 Antigenic Features, Allergenicity, and Solubility

Hsp90 antigenicity yielded scores of 0.757094 using ANTIGENpro and 0.5513 with VaxiJen, both above the threshold of 0.5. The Hsp90 allergenicity was assessed by AlgPred hybrid approach, indicating that Hsp90 is nonallergen. Also, SOLpro forecasted the protein solubility after being overexpressed in *E. coli* (likelihood: 0.62436).

## 4 Discussion

Vaccination is the most effective and rational strategy for addressing toxoplasmosis. The first step in developing an effective vaccine is the identification of parasite antigens with strong immunoprotective potential [19]. In this regard, bioinformatics‐based approaches provide valuable tools for evaluating antigenic properties and accelerating rational vaccine design. These approaches enable the prediction of protein functions, structural features, physicochemical characteristics, and potential immune epitopes, all of which are essential for antigen prioritization [48]. In this study, a detailed list of physicochemical properties relevant to the Hsp90 protein was initially forecasted using ExPASy ProtParam. Hsp90 has a sequence length of 708 amino acids and a MW of 81.93319 kDa, suggesting proper immunogenicity [49]. The instability index suggests that Hsp90 may be unstable under in vitro conditions (instability index: 43.98), which is common for large chaperone proteins and should be considered in recombinant expression and purification strategies, relatively acidic (pI: 4.96), and demonstrated thermo‐tolerance (aliphatic index: 81.50). A negative GRAVY score (−0.626) shows that it is a hydrophilic molecule, which allows for more appropriate interaction in aqueous environments. Its extinction coefficient was 68.675 M^−1^ cm^−1^ in water at 280 nm. Such biochemical properties are highly beneficial for future purification and extraction studies.

PTM sites in eukaryotic proteins are essential in choosing suitable expression hosts for recombinant protein forecasting [50]. Using NetPhos 3.1, 67 phosphorylation areas were detected within the protein sequence, which included 23 threonine, 14 tyrosine, and 30 serine residues. Nonetheless, only an acylation area was found in Hsp90. Additionally, the sequence predicted four N‐linked glycosylation areas and one O‐linked glycosylation area, indicating a moderately complex pattern of PTM. Consequently, the heterologous expression of *T. gondii* Hsp90 may require eukaryotic expression systems, such as yeast or mammalian cells, to ensure proper glycosylation and functional folding [51]. Subcellular localization analysis using DeepLoc predicted that Hsp90 is primarily localized in the cytoplasm, suggesting its involvement in cytosolic processes such as signal transduction, protein–protein interactions, and metabolic regulation. The secondary prediction of nuclear localization (score = 0.304) and the detection of a NES imply that the protein may undergo dynamic nucleocytoplasmic trafficking, a common feature observed in regulatory proteins, transcription factors, and stress‐responsive elements. Moreover, the absence of transmembrane domains and the high probability of being soluble (0.889) further support its role as a nonmembranous, cytoplasmic effector molecule, potentially functioning as a component in cytosolic signaling cascades or immune recognition pathways. Interestingly, the moderate scores for Golgi apparatus (0.3342) and lysosomal/vacuolar pathways (0.2282) cannot be disregarded, as they may hint at transient or regulated localization, possibly under stress conditions or during infection stages, especially in intracellular pathogens such as *T. gondii*. According to DEEP‐TMHMM, no transmembrane domains were found in the sequence.

Secondary structure analysis showed that Hsp90 is mainly composed of alpha helices, random coils, and extended strands. Specifically, the predicted structure included 50.85% alpha helix, 33.47% random coil, and 15.68% extended strand. These structural features are relevant to antigen‐antibody interactions, as alpha helices, beta turns, and flexible regions may contribute to epitope accessibility and conformational stability [52, 53]. In addition, two disulfide bonds were predicted using DIpro. Disulfide bonds can play important roles in maintaining protein folding, stability, and biological function [54]. Accurate prediction of disulfide bonding can reduce conformational uncertainty and improve the reliability of three‐dimensional protein modeling [55]. The Hsp90's 3D structure was modeled using the SWISS‐MODEL web server with an 82.98% identity. Following this, the model was further enhanced using the Galaxy Refine server, yielding the most refined version with favorable parameters. A lower RMSD indicates that the refined structure is similar to the original one. It is stated that “a structure with a MolProbity score lower than its crystallographic resolution is regarded as being of higher quality than an average structure at that resolution” [56, 57]. The clash score reflects steric clashes in the refined structure and is affected by the protein structure resolution. The restricted flexibility of certain residues in their side chains results in suboptimal rotamers [58].

One advantage of in silico vaccination strategies is their capacity to elicit a robust immune response that effectively targets the parasite. The process entails identifying epitopes for CTLs, B‐cells, and MHC‐binding sites of a certain protein with many online tools [59, 60]. In this study, linear B‐cell epitopes of Hsp90 were predicted using ABCpred, BCPREDS, and SVMTriP through a multistep approach. Although several predicted B‐cell epitopes showed high scores and favorable antigenicity, allergenicity analysis indicated that some ABCpred‐derived peptides may have potential allergenic properties. Therefore, antigenicity alone should not be considered sufficient for epitope prioritization. Instead, suitable vaccine candidates should ideally combine high antigenicity, good solubility, and nonallergenic characteristics. Based on these criteria, RVFIMDDCEDLIPEWL and GFSLDEPTQFAARIHR were identified as promising B‐cell epitope candidates for future experimental evaluation. In addition, 12 discontinuous B‐cell epitopes were identified using the ElliPro server, supporting the potential of Hsp90 to induce antibody‐mediated immune responses.

The peptide–MHC molecule interaction is critical for T‐cell antigen presentation and significantly affects epitope potential. Two databases were used to evaluate the IC_50_ values of peptides attached to MHC‐I and MHC‐II molecules for Hsp90. T‐cell epitopes on Hsp90 showed strong binding affinities to MHC classes I and II molecules. They identified specific peptides, and the results were largely uniform across the platforms. Lower IC_50_ values indicate increased binding affinity, indicating a better T‐cell epitope, while higher values imply decreased affinity. For MHC‐II, among all the forecasted epitopes, ANMERIMKAQALRDN (VaxiJen score: 0.7229) was found to be the most antigenic peptide. Furthermore, DKVEQVVVSNRITDS and SLTNDWEDPLAVKHF were identified as capable could induce IFN‐γ. Regarding induction, all epitopes were strong IL‐4 inducers except for HDKVEQVVVSNRITD. For MHC‐I alleles, the most immunogenic epitopes were KGAERLFIRI (PR: 1.1, immunogenicity: 0.40581), SNKEIFLREL (PR: 0.64, immunogenicity: 0.36609), and SEFISFPIEL (PR: 0.24, immunogenicity: 0.2852). The Hsp90 protein is an antigenic protein, with scores of 0.757094 and 0.5513 from ANTIGENpro and VaxiJen v2.0, respectively. The potential allergenic features of the protein were predicted by Algpred server, suggesting nonallergenicity of the protein.

A limitation of this study is that MHC binding predictions were performed exclusively using murine alleles. While murine models are widely used as a primary screening step in immunoinformatics‐based vaccine design, they do not fully represent human HLA diversity. Therefore, future studies should include HLA Class I and II alleles to improve translational relevance and vaccine applicability in humans. Another limitation of this study is the absence of epitope conservancy analysis across different strains of *T. gondii*. Since genetic variability among parasite strains may influence epitope stability and vaccine efficacy, future studies should evaluate the conservation of predicted epitopes across multiple strains to ensure broad‐spectrum protection. Additionally, human HLA allele coverage and population‐wide immunogenicity were not assessed in this study, which limits the direct extrapolation of results to human populations. Therefore, future work should incorporate HLA‐based population coverage analysis to improve translational relevance.

## 5 Conclusion

The findings highlight *T. gondii* Hsp90 as a promising target for subunit vaccine development. However, since the epitope prediction was performed using murine MHC alleles, these results are primarily applicable to mouse preclinical models. Future studies involving human HLA alleles and subsequent in vivo trials are necessary to confirm the efficacy of these epitopes in humans.

## 6 Suggestion

Employing in silico tools to forecast the function and structure of Hsp90 can decrease the risk of failure in laboratory experiments. For future studies, it is strongly recommended that in vivo or in vitro validation be performed to assess the protein's potential as a vaccine candidate.

## Author Contributions

**Amirhossein Abedi:** conceptualization, methodology, software, validation, writing – review and editing, writing – original draft. **Ali Dalir Ghaffari:** conceptualization, supervision, resources, writing – review and editing, writing – original draft, funding acquisition. **Sobhan Dehghanpour:** investigation, validation, formal analysis, writing – review and editing, writing – original draft. **Mohammad Fekrikia:** writing – original draft, writing – review and editing, project administration, resources, visualization.

## Conflicts of Interest

The authors declare no conflicts of interest.

## Supporting information


Supporting File

## Data Availability

The data set used in this study consists of the publicly available Hsp90 protein sequence retrieved from the UniProt database (Accession Number: A0A125YVB7). All computational analyses were performed using publicly available bioinformatics tools and web servers, as detailed in Table S2. No additional data sets were generated or analyzed during the current study.

## References

[1] A. Abedi and A. D. Ghaffari , “Considerations on Structural Vaccinology and Epitope Screening of Calcium‐Dependent Protein Kinases 8 as a Potential Vaccine Target Against *Toxoplasma gondii* ,” Interdisciplinary Perspectives on Infectious Diseases 2025, no. 1 (2025): 4426082. 10.1155/ipid/4426082 PMC12440659 40963642

[2] D. R. Weilhammer and A. Rasley , “Genetic Approaches for Understanding Virulence in *Toxoplasma gondii* ,” Briefings in Functional Genomics 10, no. 6 (2011): 365–373. 10.1093/bfgp/elr028 21930659

[3] Q. Liu , Z.‐D. Wang , S.‐Y. Huang , and X.‐Q. Zhu , “Diagnosis of Toxoplasmosis and Typing of T*oxoplasma gondii*,” *Parasites & Vectors* 8 (2015): 1–14. 10.1186/s13071-015-0902-6 PMC4451882 26017718

[4] A. J. L. Macario , “Heat‐Shock Proteins and Molecular Chaperones: Implications for Pathogenesis, Diagnostics, and Therapeutics,” International Journal of Clinical and Laboratory Research 25 (1995): 59–70, 10.1007/BF02592359. 7663007

[5] M. Soete , D. Camus , and J. F. Dubrametz , “Experimental Induction of Bradyzoite‐Specific Antigen Expression and Cyst Formation by the RH Strain of *Toxoplasma gondii* In Vitro,” Experimental Parasitology 78, no. 4 (1994): 361–370. 10.1006/expr.1994.1039 8206135

[6] S. Tomavo , B. Fortier , M. Soete , C. Ansel , D. Camus , and J. F. Dubremetz , “Characterization of Bradyzoite‐Specific Antigens of *Toxoplasma gondii* ,” Infection and Immunity 59, no. 10 (1991): 3750–3753. 10.1128/iai.59.10.3750-3753.1991 PMC258946 1894373

[7] M. Antczak , K. Dzitko , and H. Długońska , “Human Toxoplasmosis–Searching for Novel Chemotherapeutics,” Biomedicine & Pharmacotherapy 82 (2016): 677–684. 10.1016/j.biopha.2016.05.041 27470411

[8] G. Saadatnia and M. Golkar , “A Review on Human Toxoplasmosis,” Scandinavian Journal of Infectious Diseases 44, no. 11 (2012): 805–814. 10.3109/00365548.2012.693197 22831461

[9] S. S.‐Y. Lim and R. Y. Othman , “Recent Advances in *Toxoplasma gondii* Immunotherapeutics,” Korean Journal of Parasitology 52, no. 6 (2014): 581–593. 10.3347/kjp.2014.52.6.581 PMC4277020 25548409

[10] J. B. Alarcon , G. W. Waine , and D. P. McManus , “DNA Vaccines: Technology and Application as Anti‐Parasite and Anti‐Microbial Agents,” Advances in Parasitology 42 (1999): 343–410. 10.1016/s0065-308x(08)60152-9 10050276

[11] M. Meng , A. Zhou , G. Lu , et al., “DNA Prime and Peptide Boost Immunization Protocol Encoding the *Toxoplasma gondii* GRA4 Induces Strong Protective Immunity in BALB/c Mice,” BMC Infectious Diseases 13 (2013): 494. 10.1186/1471-2334-13-494 PMC3871000 24148219

[12] S. Dodangeh , M. Fasihi‐Ramandi , A. Daryani , R. Valadan , and S. Sarvi , “In Silico Analysis and Expression of a Novel Chimeric Antigen as a Vaccine Candidate Against *Toxoplasma gondii* ,” Microbial Pathogenesis 132 (2019): 275–281. 10.1016/j.micpath.2019.05.013 31078709

[13] M. Barros , D. Teixeira , M. Vilanova , A. Correia , N. Teixeira , and M. Borges , “Vaccines in Congenital Toxoplasmosis: Advances and Perspectives,” Frontiers in Immunology 11 (2021): 621997. 10.3389/fimmu.2020.621997 PMC7917294 33658997

[14] M.‐J. Gething and J. Sambrook , “Protein Folding in the Cell,” Nature 355, no. 6355 (1992): 33–45. 10.1038/355033a0 1731198

[15] W. B. Pratt and D. O. Toft , “Regulation of Signaling Protein Function and Trafficking by the hsp90/hsp70‐Based Chaperone Machinery,” Experimental Biology and Medicine 228, no. 2 (2003): 111–133. 10.1177/153537020322800201 12563018

[16] J. C. Young , I. Moarefi , and F. U. Hartl , “Hsp90,” Journal of Cell Biology 154, no. 2 (2001): 267–274. 10.1083/jcb.200104079 PMC2150759 11470816

[17] S. O. Angel , M. J. Figueras , M. L. Alomar , P. C. Echeverria , and B. Deng , “ *Toxoplasma gondii* Hsp90: Potential Roles in Essential Cellular Processes of the Parasite,” Parasitology 141, no. 9 (2014): 1138–1147. 10.1017/S0031182014000055 PMC4181529 24560345

[18] D. R. Flower , I. K. Macdonald , K. Ramakrishnan , M. N. Davies , and I. A. Doytchinova , “Computer Aided Selection of Candidate Vaccine Antigens,” Immunome Research 6, no. Suppl 2 (2010): S1. 10.1186/1745-7580-6-S2-S1 PMC2981880 21067543

[19] Y. Wang , G. Wang , J. Cai , and H. Yin , “Review on the Identification and Role of *Toxoplasma gondii* Antigenic Epitopes,” Parasitology Research 115, no. 2 (2016): 459–468. 10.1007/s00436-015-4824-1 26581372

[20] H. Keyvani , N. A. Ahmadi , M. M. Ranjbar , S. Ataei Kachooei , K. Ghorban , and M. Dadmanesh , “Immunoinformatics Study of gp120 of Human Immunodeficiency Virus Type 1 Subtype CRF35_AD Isolated From Iranian Patients,” Archives of Clinical Infectious Diseases 11, no. 4 (2016): e36270.

[21] P. Romano , R. Giugno , and A. Pulvirenti , “Tools and Collaborative Environments for Bioinformatics Research,” Briefings in Bioinformatics 12, no. 6 (2011): 549–561. 10.1093/bib/bbr055 PMC3220874 21984743

[22] H. Can , S. Erkunt Alak , A. E. Köseoğlu , M. Döşkaya , and C. Ün , “Do *Toxoplasma gondii* Apicoplast Proteins Have Antigenic Potential? An In Silico Study,” Computational Biology and Chemistry 84 (2020): 107158. 10.1016/j.compbiolchem.2019.107158 31810853

[23] E. Gasteiger , C. Hoogland , A. Gattiker , et al., “Protein Identification and Analysis Tools on the ExPASy Server,” in The Proteomics Protocols Handbook, ed. J. M. Walker (Humana Press, 2005), 571–607, 10.1385/1-59259-890-0:571.

[24] T. N. Petersen , S. Brunak , G. von Heijne , and H. Nielsen , “SignalP 4.0: Discriminating Signal Peptides From Transmembrane Regions,” Nature Methods 8, no. 10 (2011): 785–786. 10.1038/nmeth.1701 21959131

[25] L. Kiemer , J. D. Bendtsen , and N. Blom , “NetAcet: Prediction of N‐Terminal Acetylation Sites,” Bioinformatics 21, no. 7 (2005): 1269–1270. 10.1093/bioinformatics/bti130 15539450

[26] R. Gupta , E. Jung , and S. Brunak , *NetNGlyc 1.0 Server: Prediction of N‐Glycosylation Sites in Human Proteins* (DTU Bioinformatics, 2004).

[27] C. Steentoft , S. Y. Vakhrushev , H. J. Joshi , et al., “Precision Mapping of the Human O‐GalNAc Glycoproteome Through SimpleCell Technology,” EMBO Journal 32, no. 10 (2013): 1478–1488. 10.1038/emboj.2013.79 PMC3655468 23584533

[28] D. W. A. Buchan and D. T. Jones , “The PSIPRED Protein Analysis Workbench: 20 Years On,” Nucleic Acids Research 47, no. W1 (2019): W402–W407. 10.1093/nar/gkz297 PMC6602445 31251384

[29] J. Garnier , J.‐F. Gibrat , and B. Robson , “[32] GOR Method for Predicting Protein Secondary Structure From Amino Acid Sequence,” in Methods in Enzymology (Elsevier, 1996), 540–553. 10.1016/s0076-6879(96)66034-0 8743705

[30] M. H. Høie , E. N. Kiehl , B. Petersen , et al., “NetSurfP‐3.0: Accurate and Fast Prediction of Protein Structural Features by Protein Language Models and Deep Learning,” Nucleic Acids Research 50, no. W1 (2022): W510–W515. 10.1093/nar/gkac439 PMC9252760 35648435

[31] A. Waterhouse , M. Bertoni , S. Bienert , et al., “SWISS‐MODEL: Homology Modelling of Protein Structures and Complexes,” Nucleic Acids Research 46, no. W1 (2018): W296–W303. 10.1093/nar/gky427 PMC6030848 29788355

[32] J. Ko , H. Park , L. Heo , and C. Seok , “GalaxyWEB Server for Protein Structure Prediction and Refinement,” Nucleic Acids Research 40, no. W1 (2012): W294–W297. 10.1093/nar/gks493 PMC3394311 22649060

[33] T. Nugent , D. Cozzetto , and D. T. Jones , “Evaluation of Predictions in the CASP10 Model Refinement Category,” Proteins: Structure, Function, and Bioinformatics 82 (2014): 98–111. 10.1002/prot.24377 PMC4282348 23900810

[34] M. Bertoni , F. Kiefer , M. Biasini , L. Bordoli , and T. Schwede , “Modeling Protein Quaternary Structure of Homo‐and Hetero‐Oligomers Beyond Binary Interactions by Homology,” Scientific Reports 7, no. 1 (2017): 10480. 10.1038/s41598-017-09654-8 PMC5585393 28874689

[35] A. Narula , R. K. Pandey , N. Khatoon , A. Mishra , and V. K. Prajapati , “Excavating Chikungunya Genome to Design B and T Cell Multi‐Epitope Subunit Vaccine Using Comprehensive Immunoinformatics Approach to Control Chikungunya Infection,” Infection, Genetics and Evolution 61 (2018): 4–15. 10.1016/j.meegid.2018.03.007 29535024

[36] S. Saha and G. P. S. Raghava , “Prediction of Continuous B‐Cell Epitopes in an Antigen Using Recurrent Neural Network,” Proteins: Structure, Function, and Bioinformatics 65, no. 1 (2006): 40–48. 10.1002/prot.21078 16894596

[37] N. Kumar , S. Tripathi , N. Sharma , S. Patiyal , N. L. Devi , and G. P. S. Raghava , “A Method for Predicting Linear and Conformational B‐Cell Epitopes in an Antigen From Its Primary Sequence,” Computers in Biology and Medicine 170 (2024): 108083. 10.1016/j.compbiomed.2024.108083 38295479

[38] S. Saha and G. P. S. Raghava , “BcePred: Prediction of Continuous B‐Cell Epitopes in Antigenic Sequences Using Physico‐Chemical Properties,” in International Conference on Artificial Immune Systems (Springer, 2004).

[39] Y. EL‐Manzalawy , D. Dobbs , and V. Honavar , “Predicting Linear B‐Cell Epitopes Using String Kernels,” Journal of Molecular Recognition 21, no. 4 (2008): 243–255. 10.1002/jmr.893 PMC2683948 18496882

[40] B. Yao , L. Zhang , S. Liang and C. Zhang . “SVMTriP: A Method to Predict Antigenic Epitopes Using Support Vector Machine to Integrate Tri‐Peptide Similarity and Propensity,” PLoS One 7, no. 9 (2012): e45152, 10.1371/journal.pone.0045152. PMC3440317 22984622

[41] A. D. Ghaffari , A. Dalimi , F. Ghaffarifar , and M. Pirestani , “Antigenic Properties of Dense Granule Antigen 12 Protein Using Bioinformatics Tools in Order to Improve Vaccine Design Against *Toxoplasma gondii* ,” Clinical and Experimental Vaccine Research 9, no. 2 (2020): 81. 10.7774/cevr.2020.9.2.81 PMC7445328 32864364

[42] S. Saha and G. P. S. Raghava , “AlgPred: Prediction of Allergenic Proteins and Mapping of IgE Epitopes,” Nucleic Acids Research 34, no. suppl_2 (2006): W202–W209. 10.1093/nar/gkl343 PMC1538830 16844994

[43] J. J. A. Calis , M. Maybeno , J. A. Greenbaum , et al., “Properties of MHC Class I Presented Peptides That Enhance Immunogenicity,” PLoS Computational Biology 9, no. 10 (2013): e1003266. 10.1371/journal.pcbi.1003266 PMC3808449 24204222

[44] M. Bhasin and G. P. S. Raghava , “Prediction of CTL Epitopes Using QM, SVM and ANN Techniques,” Vaccine 22, no. 23–24 (2004): 3195–3204. 10.1016/j.vaccine.2004.02.005 15297074

[45] C. N. Magnan , M. Zeller , M. A. Kayala , et al., “High‐Throughput Prediction of Protein Antigenicity Using Protein Microarray Data,” Bioinformatics 26, no. 23 (2010): 2936–2943. 10.1093/bioinformatics/btq551 PMC2982151 20934990

[46] I. A. Doytchinova and D. R. Flower , “VaxiJen: A Server for Prediction of Protective Antigens, Tumour Antigens and Subunit Vaccines,” BMC Bioinformatics 8, no. 1 (2007): 4. 10.1186/1471-2105-8-4 PMC1780059 17207271

[47] C. N. Magnan , A. Randall , and P. Baldi , “SOLpro: Accurate Sequence‐Based Prediction of Protein Solubility,” Bioinformatics 25, no. 17 (2009): 2200–2207. 10.1093/bioinformatics/btp386 19549632

[48] N.‐Z. Zhang , J. Chen , M. Wang , E. Petersen , and X. Q. Zhu , “Vaccines Against *Toxoplasma gondii*: New Developments and Perspectives,” Expert Review of Vaccines 12, no. 11 (2013): 1287–1299. 10.1586/14760584.2013.844652 24093877

[49] H. Majidiani , S. Soltani , A. D. Ghaffari , M. Sabaghan , A. Taghipour , and M. Foroutan , “In‐Depth Computational Analysis of Calcium‐Dependent Protein Kinase 3 of *Toxoplasma gondii* Provides Promising Targets for Vaccination,” Clinical and Experimental Vaccine Research 9, no. 2 (2020): 146. 10.7774/cevr.2020.9.2.146 PMC7445322 32864371

[50] M. Hansson , P. A. k. Nygren , and S. Sta˚hl , “Design and Production of Recombinant Subunit Vaccines,” Biotechnology and Applied Biochemistry 32, no. 2 (2000): 95–107. 10.1042/ba20000034 11001870

[51] M. Shams , E. Javanmardi , M. C. Nosrati , et al., “Bioinformatics Features and Immunogenic Epitopes of *Echinococcus granulosus* Myophilin as a Promising Target for Vaccination Against Cystic Echinococcosis,” Infection, Genetics and Evolution 89 (2021): 104714. 10.1016/j.meegid.2021.104714 33434702

[52] M. Shaddel , M. Ebrahimi , and M. R. Tabandeh , “Bioinformatics Analysis of Single and Multi‐Hybrid Epitopes of GRA‐1, GRA‐4, GRA‐6 and GRA‐7 Proteins to Improve DNA Vaccine Design Against *Toxoplasma gondii* ,” Journal of Parasitic Diseases 42, no. 2 (2018): 269–276. 10.1007/s12639-018-0996-9 PMC5962505 29844632

[53] G. Yadav , R. Rao , U. Raj , and P. K. Varadwaj , “Computational Modeling and Analysis of Prominent T‐Cell Epitopes for Assisting in Designing Vaccine of ZIKA Virus,” Journal of Applied Pharmaceutical Science 7, no. 8 (2017): 116–122.

[54] J. R. Winther and C. Thorpe , “Quantification of Thiols and Disulfides,” Biochimica et Biophysica Acta (BBA)–General Subjects 1840, no. 2 (2014): 838–846. 10.1016/j.bbagen.2013.03.031 PMC3766385 23567800

[55] J. Yang , B. J. He , R. Jang , Y. Zhang , and H. B. Shen , “Accurate Disulfide‐Bonding Network Predictions Improve Ab Initio Structure Prediction of Cysteine‐Rich Proteins,” Bioinformatics 31, no. 23 (2015): 3773–3781. 10.1093/bioinformatics/btv459 PMC5898604 26254435

[56] J. Chen , H. Liu , J. Yang , and K. C. Chou , “Prediction of Linear B‐Cell Epitopes Using Amino Acid Pair Antigenicity Scale,” Amino Acids 33, no. 3 (2007): 423–428. 10.1007/s00726-006-0485-9 17252308

[57] V. B. Chen , W. B. Arendall , J. J. Headd , et al., “MolProbity: All‐Atom Structure Validation for Macromolecular Crystallography,” Acta Crystallographica Section D Biological Crystallography 66, no. 1 (2010): 12–21. 10.1107/S0907444909042073 PMC2803126 20057044

[58] M. Sasai and M. Yamamoto , “Innate, Adaptive, and Cell‐Autonomous Immunity Against *Toxoplasma gondii* Infection,” Experimental & Molecular Medicine 51, no. 12 (2019): 1–10. 10.1038/s12276-019-0353-9 PMC6906438 31827072

[59] M. Foroutan , F. Ghaffarifar , Z. Sharifi , and A. Dalimi , “Vaccination With a Novel Multi‐Epitope ROP8 DNA Vaccine Against Acute *Toxoplasma gondii* Infection Induces Strong B and T Cell Responses in Mice,” Comparative Immunology, Microbiology and Infectious Diseases 69 (2020): 101413. 10.1016/j.cimid.2020.101413 31954995

[60] M. Foroutan , F. Ghaffarifar , Z. Sharifi , A. Dalimi , and M. Pirestani , “Bioinformatics Analysis of ROP8 Protein to Improve Vaccine Design Against *Toxoplasma gondii* ,” Infection, Genetics and Evolution 62 (2018): 193–204. 10.1016/j.meegid.2018.04.033 29705360

