# Supplementary material for: Computational Prediction of Immunogenic Epitopes and Protein Structures of Heat Shock Protein 90 in Toxoplasma gondii
Source: Immun Inflamm Dis. 2026 Nov 6;14(11):e70584. doi: 10.1002/iid3.70584 (PMC13741397; doi:10.1002/iid3.70584)
Supplement: Supplementary file 1 [file IID3-14-e70584-s001.docx]

**Supplementary Table 1.** The links of all bioinformatics online servers used in the research

| **Parameter** | **Value** | **Interpretation / Description** |
| --- | --- | --- |
| Protein name | TgHsp90 | Heat shock protein 90 of *Toxoplasma gondii* |
| UniProt accession | A0A125YVB7 | Sequence used for ProtParam analysis |
| Number of amino acids | 708 | Protein length |
| Molecular weight | 81.93319 kDa | Predicted molecular mass |
| Theoretical pI | 4.96 | Indicates acidic nature |
| Instability index | 43.98 | Protein predicted as unstable because value > 40 |
| Aliphatic index | 81.50 | Indicates relative volume occupied by aliphatic side chains |
| GRAVY | -0.626 | Negative value indicates hydrophilic character |
| Prediction tool | ExPASy ProtParam | Physicochemical property prediction |
| Input sequence | TgHsp90 protein sequence | UniProt accession A0A125YVB7 |

**Supplementary Table 2.** The links of all bioinformatics online servers used in the research

| Online site | Link |
| --- | --- |
| UniProtKB | <https://www.uniprot.org> |
| Expasy ProtParam | <https://web.expasy.org/protparam/> |
| SignalP | <https://services.healthtech.dtu.dk/services/SignalP-5.0/> |
| LipoP | <https://services.healthtech.dtu.dk/services/LipoP-1.0/> |
| TatP | <https://services.healthtech.dtu.dk/services/TatP-1.0/> |
| NetAcet | <https://services.healthtech.dtu.dk/services/NetAcet-1.0/> |
| VaxiJen v. 2.0 | <http://www.ddg-pharmfac.net/vaxijen/VaxiJen/VaxiJen.html> |
| AlgPred | <https://webs.iiitd.edu.in/raghava/algpred/submission.html> |
| SOLpro | [https://scratch.proteomics.ics.uci.edu/explanation.html#SOLpro](https://scratch.proteomics.ics.uci.edu/explanation.html%23SOLpro) |
| NetPhos 3.1 | <http://www.cbs.dtu.dk/services/NetPhos/> |
| NetOGlyc 4.0 | <http://www.cbs.dtu.dk/services/NetOGlyc/> |
| NetNGlyc 1.0 | <http://www.cbs.dtu.dk/services/NetNGlyc/> |
| DeepLoc-2.1 | <https://services.healthtech.dtu.dk/services/DeepLoc-2.1/> |
| DeepTMHMM-1.0 | <https://services.healthtech.dtu.dk/services/DeepTMHMM-1.0/> |
| GOR IV | <https://npsa-prabi.ibcp.fr/cgi-bin/npsa_automat.pl?page=npsa_gor4.html> |
| PSIPRED | <http://bioinf.cs.ucl.ac.uk/psipred/> |
| NetSurfP3 | <https://services.healthtech.dtu.dk/services/NetSurfP-3.0/> |
| DIpro | <http://scratch.proteomics.ics.uci.edu/> |
| GalaxyRefine | <http://galaxy.seoklab.org/cgi-bin/submit.cgi?type=REFINE> |
| SWISS-MODEL | <https://swissmodel.expasy.org/> |
| Prosa-Web | <https://prosa.services.came.sbg.ac.at/prosa.php> |
| ABCpred | <http://crdd.osdd.net/raghava/abcpred/> |
| Bcepred | <http://crdd.osdd.net/raghava/bcepred/bcepred_submission.html> |
| BCPREDS 1.0 | <http://ailab-projects2.ist.psu.edu/bcpred/predict.html> |
| SVMTriP | <http://sysbio.unl.edu/SVMTriP/> |
| ProtScale | <https://web.expasy.org/protscale/> |
| ElliPro | <http://tools.iedb.org/ellipro> |
| IEDB | <http://tools.iedb.org/bcell/> |
| PepCalc | <https://pepcalc.com/> |
| IEDB MHCI | <http://tools.iedb.org/mhci/> |
| IEDB MHCII | <http://tools.iedb.org/mhcii/> |
| Immunogenicity | <http://tools.iedb.org/immunogenicity/> |
| IFNepitope | <https://webs.iiitd.edu.in/raghava/ifnepitope/predict.php> |
| IL4-pred | <https://webs.iiitd.edu.in/raghava/il4pred/design.php> |
| CTLpred | <http://crdd.osdd.net/raghava/ctlpred/about.html> |
| ANTIGENpro | <https://scratch.proteomics.ics.uci.edu/> |
